# Can Leukocyte Morphology Contribute to Welfare Assessment in Beef Cattle Raised Under Intensive and Extensive Farming Systems?

**DOI:** 10.3390/ani16213356

**Published:** 2026-10-24

**Authors:** Laura Raquel Rios Ribeiro, Juliana do Canto Olegário, Alexandre de Oliveira Bezerra, Ricardo Martins Santos, Cesar Augusto Taconeli, Carla Forte Maiolino Molento

**Affiliations:** 1 Animal Welfare Laboratory, Department of Animal Science, Federal University of Paraná, Curitiba 80035-050, Brazilcarlamolento@ufpr.br (C.F.M.M.); 2 Department of Veterinary Medicine, Dom Bosco Catholic University, Campo Grande 79117-900, Brazil; 3 Prime College—Prime Education Group, Campo Grande 79118-130, Brazil; 4 Department of Statistics, Federal University of Paraná, Curitiba 81531-980, Brazil

**Keywords:** animal welfare, beef cattle, hematological biomarkers, leukocyte morphology, neutrophil morphology, lymphocyte morphology, welfare assessment

## Abstract

To investigate the potential contribution of leukocyte morphology to animal welfare assessment, this study evaluated leukocyte profiles in beef cattle raised under intensive and extensive production systems. A total of 664 blood samples were collected at the end of the production cycle from commercial farms in Mato Grosso do Sul, Brazil. Welfare assessments included qualitative behavioral assessment, health indicators, hematocrit, total and differential leukocyte counts, neutrophil-to-lymphocyte (N/L) ratio, and leukocyte morphology using an image-supported scoring system. In pooled descriptive data, severe neutrophil morphological alterations were observed in 27.1% of cattle from intensive systems and 11.4% from extensive systems, while atypical lymphocytes were observed in 21.8% and 9.6%, respectively. Exploratory season-specific generalized estimating equation (GEE) analyses indicated higher neutrophil counts and N/L ratios in cattle from intensive systems in both autumn and spring. In autumn, lymphocyte counts were lower in cattle from the intensive system, and severe neutrophil morphological alterations and atypical lymphocytes had higher estimated probabilities. In spring, no clear evidence of differences between systems was observed for lymphocyte counts or leukocyte morphology. Qualitative behavioral assessment revealed variation among farms, including farms within the same production system. Health alterations, including nasal discharge, coughing, and swelling, were recorded exclusively in the evaluated intensive systems. These findings indicate differences in leukocyte morphology among cattle from the evaluated farms and suggest that leukocyte morphological assessment may provide a useful complementary animal-based indicator within multidimensional welfare assessment frameworks.

## Simple Summary

Animal welfare is influenced by health, behavior, and the conditions in which animals live. Identifying practical indicators capable of reflecting how animals feel in different production environments remains an important challenge for animal welfare science. Leukocytes are routinely used to evaluate health and immune responses, and their counts eventually are used to assess animal welfare; however, little attention has been given to changes in their morphology as potential indicators of welfare. In this study, we compared beef cattle raised in intensive and extensive production systems and evaluated both leukocyte counts and morphology using an image-supported scoring system. Cattle from the evaluated intensive farms showed more frequent leukocyte morphological alterations, while health and behavioral indicators revealed differences among farms. These findings may reflect variations in environmental, health, and management conditions, rather than effects attributable exclusively to the production system. The results suggest that the microscopic evaluation of leukocyte morphology may provide additional information about cattle welfare status in different production conditions. Because blood smear evaluation is inexpensive and routinely performed in veterinary medicine, the fact that leukocyte morphology may represent a useful complementary tool for animal welfare assessment seems of practical relevance.

## 1. Introduction

Brazil has one of the largest cattle populations worldwide, with approximately 195.5 million animals, corresponding to 12% of the global cattle population. The country produces approximately 12.35 million tonnes of beef annually, representing 16.1% of global production, and accounts for approximately 36.7% of internationally traded beef according to the Beef Report 2026 [1]. Beyond their economic significance, these figures represent a vast number of individual animal lives and reinforce the importance of understanding the welfare implications of the diverse production systems used in the country [2,3]. Understanding how animal production systems affect animals is also relevant to the development of more ethical and sustainable agricultural practices [4].

Farm animal welfare has received increasing scientific, ethical, and societal attention over recent decades [5]. This concern is supported by the recognition of animals as sentient beings capable of experiencing positive and negative affective states and by the growing understanding of animal welfare as an essential component of responsible and sustainable animal production systems [6,7,8,9]. Within this perspective, animal welfare is increasingly understood as part of social sustainability, requiring food systems to consider animals not only as production units but as sentient individuals with their own interests and welfare needs [7,10,11]. This development is also reflected in the progressive incorporation of animal protection and welfare principles into international policies, guidelines, and action plans [12,13].

Animal welfare science has been described as a science with a societal mandate because it is guided both by scientific questions and by ethical concerns regarding how animals experience the conditions in which they live [14]. It seeks to understand animals’ physical, behavioral, psychological, and social needs and to provide evidence that supports the prevention of suffering and the promotion of appropriate living conditions [15,16]. Within this framework, accurate welfare assessment is a central priority and has stimulated the search for reliable and quantitative indicators that can complement existing welfare assessment approaches [17]. Because animal welfare is multidimensional, it cannot be adequately characterized by a single measure. Its assessment therefore requires the integration of animal-based, resource-based, and management-based indicators [18,19]. Animal-based indicators are particularly relevant because they reflect how individual animals respond to the conditions to which they are exposed.

Physiological stress responses are closely related to animal welfare, although their interpretation depends on the nature, intensity, duration, predictability, and controllability of the challenge. Changes in affective state and exposure to demanding conditions may activate the hypothalamic–pituitary–adrenal axis and other neuroendocrine pathways, producing physiological adjustments that support short-term coping [20,21]. However, when such activation is repeated or prolonged, it may disrupt homeostasis, alter immune function, increase disease susceptibility, and compromise welfare [22,23].

Physiological indicators, particularly those related to immune function, have therefore been investigated as complementary tools for assessing animals’ biological responses to environmental and management challenges [23,24,25]. Leukocyte numbers and relative proportions may change in response to inflammation, infection, neuroendocrine activation, and other physiological challenges, providing potentially relevant information for welfare assessment when interpreted alongside behavioral, health, and environmental measures [22,26,27]. Previous studies in cattle support the potential relevance of leukocyte-based indicators. In one study, a lower prevalence of WC1+ γδ T lymphocytes was reported in feedlot cattle than in pasture-raised cattle [28]. In dairy cows, housing conditions and access to pasture have also been associated with total lymphocyte counts and other hematological variables [29,30].

Total leukocyte count and the neutrophil-to-lymphocyte (N/L) ratio have received increasing attention as potential indicators of stress, health status, and animal welfare in cattle [30,31]. However, quantitative measurements alone may not capture all biologically relevant changes in circulating leukocytes. Unlike cell counts and ratios, morphological alterations may provide additional information regarding leukocyte maturation, activation and the presence of inflammatory or antigenic stimulation at the individual level. Neutrophil alterations such as cytoplasmic basophilia, vacuolization, abnormal granulation, nuclear hyposegmentation, and the presence of immature forms may occur in association with accelerated granulopoiesis and systemic inflammatory responses, whereas reactive lymphocyte morphology may indicate antigenic stimulation [32,33].

Despite the established diagnostic relevance of leukocyte morphology in veterinary clinical pathology, these characteristics have received little attention in animal welfare research. Most cattle welfare studies using immune or hematological measures have focused on dairy cattle or on quantitative cell counts and ratios [28,29,34,35]. To our knowledge, no previous study has systematically compared neutrophil and lymphocyte morphology in beef cattle raised under intensive and extensive systems or implemented an image-supported cell scoring system for this purpose.

In Brazil, beef cattle are raised under both extensive pasture-based and intensive feedlot systems. The systems differ in space allowance, feeding, human contact, environmental exposure, social conditions, and opportunities for behavioral expression, potentially imposing distinct physical and affective challenges on animals [7,9,36]. Extensive pasture-based systems generally provide greater space and opportunities for locomotion and species-specific behavior but may expose cattle to climatic extremes, variable food availability, parasites, and less frequent human supervision [37]. Intensive systems may provide closer control of nutrition, water provision, and health management, while potentially exposing animals to restricted movement, higher stocking densities, altered social environments, and more frequent handling [36,38]. Transitions from pasture to confinement have been associated with behavioral and physiological responses indicative of challenge, whereas access to pasture has been associated with favorable welfare and hematological outcomes in some cattle populations [34,39]. However, evidence regarding the welfare consequences of different production systems is not uniform, because outcomes depend on management practices, resource availability, environmental conditions, health status, and the experiences of individual animals. Thus, production-system labels alone do not determine welfare outcomes, although the contrasting conditions associated with intensive and extensive systems may generate different physiological and immunological responses.

Given the distinct environmental and management conditions associated with intensive and extensive production systems, the combined evaluation of leukocyte counts and morphology may provide additional information on the physiological responses of cattle relevant to welfare assessment [23]. We hypothesized that beef cattle raised under contrasting intensive and extensive finishing systems would exhibit different leukocyte profiles, including leukocyte counts, the N/L ratio, and neutrophil and lymphocyte morphology. We further hypothesized that an image-supported leukocyte scoring system would allow morphological alterations to be systematically identified and classified. Therefore, this study aimed to investigate leukocyte counts and morphological characteristics in beef cattle raised under intensive and extensive production systems and to explore their potential contribution to animal welfare assessment. Specifically, we aimed to: (1) compare total and differential leukocyte counts, the N/L ratio, and the morphological characteristics of neutrophils and lymphocytes between cattle from the two production systems; and (2) implement an image-supported cell scoring system for the recognition and classification of leukocyte morphological alterations.

## 2. Materials and Methods

### 2.1. Description of Studied Farms, Data Collection and Sample Sizes

**Figure 1:**
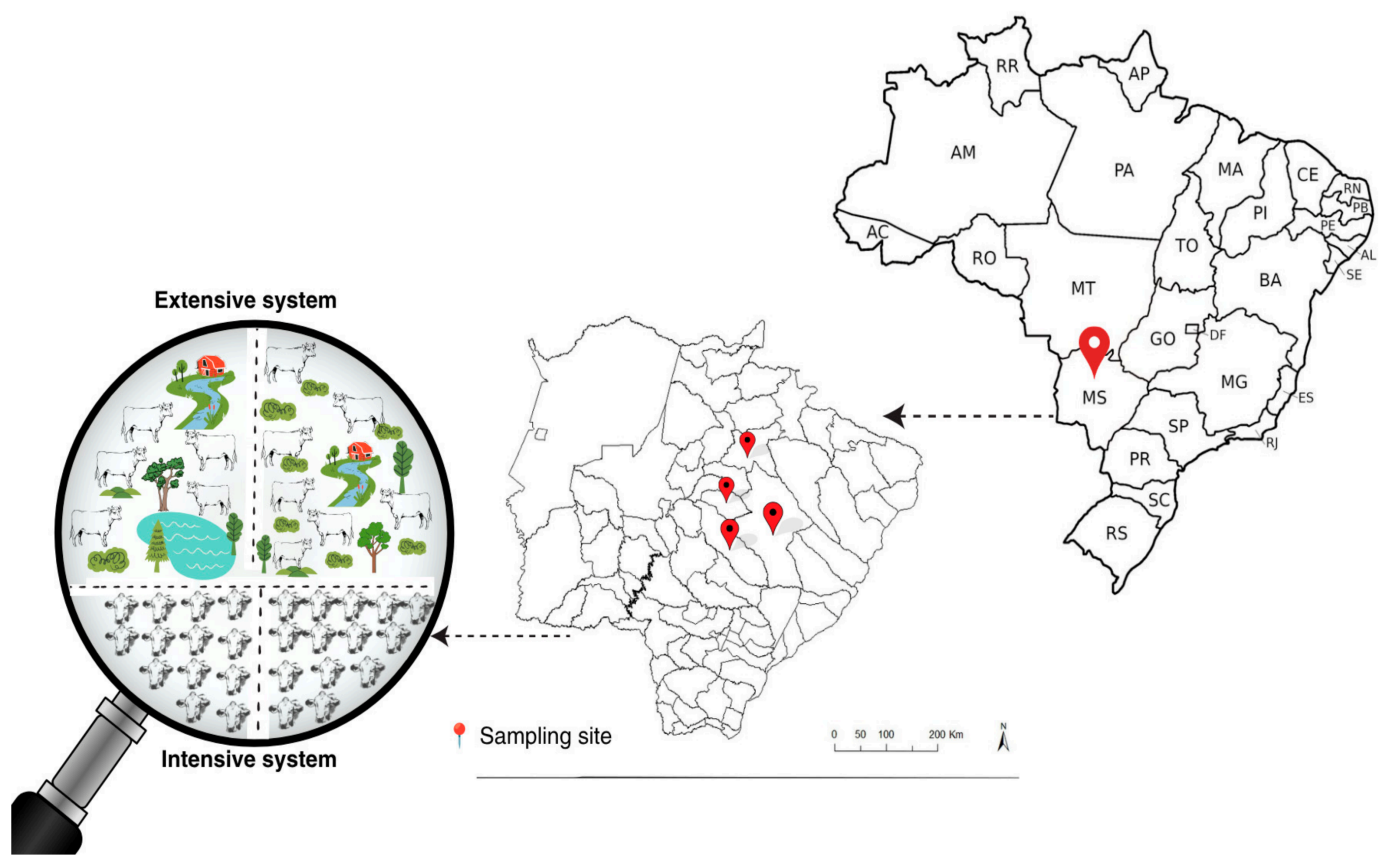
Location of the study farms in Mato Grosso do Sul, Brazil. Red markers indicate the sampling sites, and the magnified section illustrates the intensive and extensive production systems included in the study.

Field data collection was conducted on four commercial beef cattle farms situated in the municipalities of Jaraguari, Ribas do Rio Pardo, Campo Grande and Camapuã, in the state of Mato Grosso do Sul, Brazil (Figure 1). Two farms operated under intensive feedlot systems and two under extensive pasture-based systems. Animal procedures were approved by the Animal Use Ethics Committee of the Federal University of Paraná (protocol 023/2022). Representative images of the intensive and extensive farming environments are provided in Supplementary Figures S1 and S2, respectively.

Data collection was conducted during two seasonal periods. In autumn, one intensive and one extensive farm were assessed, whereas the other intensive and extensive farms were assessed in spring. Thus, both production systems were represented in each sampling season.

Field activities on each farm were organized into three main assessment stages: farm characterization and familiarization with management practices, on-farm welfare assessment, and blood sampling. Depending on farm logistics and management schedules, completion of these stages required approximately three to six visits per farm. Ambient temperature and relative humidity were recorded during these repeated visits using an Akso AZ77535 thermo-hygrometer (Akso, São Leopoldo, Rio Grande do Sul, Brazil). The retained environmental records were summarized by sampling season rather than by individual farm. During autumn, recorded temperatures ranged from approximately 20.0 to 24.0 °C and relative humidity from 55 to 76%, whereas during spring temperatures ranged from approximately 28.0 to 35.0 °C and relative humidity from 55 to 76%.

A questionnaire and herd records were used to obtain general farm information, including initial cattle numbers, herd size at the time of the visit, breed, age and mortality, and culling rates (Table 1). Additionally, specific parameters regarding herd health management, such as vaccination and parasite control protocols, were recorded (Table 2). Information on recent antimicrobial or other therapeutic treatments was not available and therefore was not incorporated into the analysis. The participating farms raised male crossbred Nellore cattle during the finishing phase. Cattle were evaluated at 20 to 24 months of age weighing an average of 440 kg, and assessments were conducted 5 ± 3 days prior to slaughter.

The assessments on each farm were carried out on three different occasions. The first visit was dedicated to farm characterization and familiarization with management practices. The second visit involved the application of welfare assessments based on selected measures from the Welfare Quality^®^ [40] protocol for cattle. During the third visit, blood samples were collected for hematological and leukocyte morphology analyses following standard procedures [32,41].

During the visit, a partial on-farm welfare assessment was conducted by two assessors using the Welfare Quality^®^ protocol for cattle [40], with the selected measures of body condition score, cleanliness, integument alterations, lameness, human-animal relationship, and Qualitative Behavior Assessment (QBA). The on-farm welfare assessment procedures are illustrated in Supplementary Figure S4. The assessors were trained and had prior experience applying the protocol; both assessors participated in all visits. The blood samples were collected after the assessment of all other indicators, during routine handling procedures (weighing, vaccination, deworming or loading), always in the morning, at the same time of day (6am to 11am), to minimize any impact of circadian variation in blood parameters when comparing farms. Animals were not subjected to fasting specifically for blood collection and remained under the routine feeding management of each farm. On the sampling day, cattle were brought from their respective pens or pastures to the handling facilities in groups, and blood was collected shortly after each animal entered the restraint chute, without additional experimental handling or prolonged individual restraint before sampling. When sampling coincided with vaccination or deworming, blood was collected before administration of vaccines or anthelmintic treatments. A standardized interval between the last feeding or previous handling event and blood collection was not recorded.

For the sample collection procedure, each animal was restrained following standard recommendations of [32,41]. A total of 664 blood samples were collected for this study. The sample size was determined using the same statistical approach previously applied to broiler chickens based on the mean and standard deviation (SD) of the heterophil-lymphocyte (H/L) ratio [42]. The formula used was *n* = 2 × *SD*^2^ × (*Z*_α/2_ + *Z*_β_)^2^/d^2^, where SD is the standard deviation from previous or pilot studies, Z_α/2_ is the critical value for the desired confidence level, Z_β_ corresponds to the power of the statistical test, and d represents the expected effect size. For this study, we adopted a 95% confidence level (Z_α/2_ = 1.96) and a statistical power of 90% (Z_β_ = 1.282).

### 2.2. Blood Sample Collection

Approximately 5 mL of blood was drawn from the coccygeal vein put into sterilized vacuum tubes (Vacutainer^®^, Becton, Dickinson and Company, Franklin Lakes, New Jersey, USA) containing ethylene diamine tetra-acetic acid (K_2_EDTA) to prevent blood clotting. Samples were then transported in an insulated container to the hematology laboratories at the Federal University of Mato Grosso do Sul and the Dom Bosco Catholic University in Campo Grande, MS, for processing and analysis. The blood collection and sample handling procedures are illustrated in Supplementary Figure S3.

### 2.3. Qualitative Behavioral Assessment of Cattle Affective States

The Qualitative Behavior Assessment (QBA) was performed on all farms during the on-farm welfare assessment stage, before animal restraint and blood sampling. This qualitative assessment considers the body language of the beef cattle, particularly the expressive quality of how cattle behave and interact with each other and their environment (43). The herds were assessed according to the Welfare Quality^®^ protocol for cattle [40], and 20 behavioral expressions were scored using a visual analogue scale (VAS). The absence of each behavioral expression was coded in blue, whereas its maximum perceived expression was coded in red.

The behavioral expressions evaluated were relaxed, sociable, positively occupied, satisfied, active, friendly, calm, inquisitive, lively, happy, uneasy, distressed, uncomfortable, frustrated, indifferent, irritable, tense, bored, nervous, and resistant [40,43,44]. The QBA was conducted at the group level by two trained assessors, who performed the evaluations simultaneously and independently, without communication during scoring.

### 2.4. Selected On-Farm Welfare Indicators

On-farm welfare assessment was conducted using selected animal- and resource-based measures derived from the Welfare Quality^®^ protocol for fattening cattle [40]. Environmental assessment included cleanliness of water points, space allowance or stocking rate, and ambient temperature and relative humidity recorded during farm visits. Air quality was not quantitatively assessed. The selected welfare indicators, assessment methods, and corresponding scoring criteria are detailed in Supplementary Table S1.

Nutritional status was evaluated individually using the Body Condition Score (BCS), classified as normal or severe change. As a resource-based indicator, cleanliness of water points was assessed visually at the time of inspection following the Welfare Quality^®^ protocol for fattening cattle [40]. Water points were classified as score 0 (clean: drinker and water clean), score 1 (partly dirty: drinker dirty but water fresh and clean), or score 2 (dirty: both drinker and water dirty at the time of inspection). Animal cleanliness was visually inspected on individual animals and classified as normal (less than 25% of the body surface covered with dirt plaques or less than 50% with liquid dirt) or severe change (greater than 25% of the body surface covered with plaques or greater than 50% with liquid dirt).

Health indicators were assessed using selected measures from the Welfare Quality^®^ protocol for cattle [40], including lameness, integument alterations (hairless spots, lesions, and swellings), coughing, nasal and ocular discharge, hampered respiration, diarrhoea, and bloated rumen. These indicators were recorded using the corresponding predefined scoring criteria (Score 0: absence or normal condition; Score 2: presence or severe alteration). Swelling in the prepuce region was also recorded when observed. Microbiological or bacterial etiological testing was not performed. Specific inflammatory biomarkers, diagnostic testing for infectious diseases, and etiological diagnoses were not included in the study. The health indicators were assessed as part of the welfare evaluation following the Welfare Quality^®^ protocol [40] and were not intended to establish etiological diagnoses. Animals presenting clinical abnormalities were retained in the hematological and morphological analyses, and health status was not included as a covariate in the statistical analysis.

The human–animal relationship was assessed using the avoidance distance test, following the Welfare Quality^®^ protocol for fattening cattle [40], during the on-farm welfare assessment stage and before animal restraint and blood sampling. An attentive animal was approached from the front, starting at approximately 3 m whenever possible. The assessor approached at approximately one step per second, with a step length of approximately 60 cm and the arm extended at approximately 45° from the body, with the back of the hand directed toward the animal. The assessor directed their gaze toward the animal’s muzzle and continued the approach until the animal showed a withdrawal response or until the muzzle could be touched.

Withdrawal was defined as the animal moving backward, turning its head away, pulling its head back, or otherwise clearly withdrawing from the approaching assessor. The avoidance distance was recorded as the distance between the assessor’s hand and the animal’s muzzle at the moment of withdrawal. Animals were classified as score 0 when the assessor could touch the animal, score 1 when the assessor could approach closer than 50 cm but could not touch the animal, score 2 when the avoidance distance was between 50 and 100 cm, and score 3 when the assessor could not approach within 100 cm [40].

### 2.5. Blood Analysis

#### 2.5.1. Blood Smears

Blood smears were prepared immediately after collection using the spreader-slide technique. Once air-dried, the smears were fixed and stained using Wright-Giemsa solution (PA202, Newprov, Pinhais, Paraná, Brazil), following the manufacturer’s instructions.

#### 2.5.2. Hematocrit (HCT) Determination

In the laboratory, blood samples in K_2_EDTA tubes were homogenized by gentle inversion. Capillary tubes were filled with blood, sealed and centrifuged in a microhematocrit centrifuge (Cm-12000–220V, Daiki, Jiangsu Jinyi Instrument Technology Co., Ltd., Changzhou, China) at 15,300× *g* for 5 min. The hematocrit (HCT) was determined using a microhematocrit reader card. For clinical classification, HCT values within the reference range for beef cattle were scored as 0 (normal), whereas values falling either below or above the reference thresholds were scored as 1 (abnormal).

#### 2.5.3. Total Leukocyte Count (TLC)

Total leukocyte count (TLC) was performed manually using a Neubauer hemocytometer (Perfecta, São Paulo, São Paulo, Brazil). Blood samples were diluted 1:20 in Turk’s solution (Newprov, Pinhais, Paraná, Brazil) to lyse erythrocytes and stain leukocyte nuclei. The diluted samples were loaded into the chamber, and leukocytes were counted under light microscopy (100× magnification) across the four large corner squares. The final TLC (cells/µL) was calculated based on the total count, dilution factor and the chamber volume: TLC (cells/µL) = (N × dilution factor)/counted chamber volume, where N is the total number of leukocytes counted, the dilution factor was 20, and the total counted chamber volume was 0.4 µL. Thus, TLC (cells/µL) = N × 20/0.4 = N × 50. Values were reported as ×10^3^/µL.

#### 2.5.4. Differential Leukocyte Counts and Cell Scoring System

Differential leukocyte counts were performed on stained blood smears using an Axio Scope A1 optical microscope (Carl Zeiss Microscopy GmbH, Jena, Germany) equipped with ZEN Lite (Blue Edition) imaging software at 1000× magnification. One hundred leukocytes were counted per slide in at least 10 microscopic fields and classified as segmented neutrophils, immature neutrophils, lymphocytes, eosinophils, basophils, or monocytes. Immature neutrophils included band neutrophils, metamyelocytes, and myelocytes. Images of the blood smears were obtained using an Axiocam 503 color camera (Carl Zeiss Microscopy GmbH, Jena, Germany) connected to the microscope. Morphologically altered neutrophils and lymphocytes were included in the differential leukocyte count. Neutrophil morphology was classified according to previously described morphological criteria [32,33].

An image-supported cell scoring system was used for the recognition and classification of morphological alterations in bovine neutrophils and lymphocytes. Neutrophil scores ranged from 0 to 4, with normal segmented neutrophils classified as score 0, band neutrophils as score 1, metamyelocytes as score 2, myelocytes as score 3, and neutrophils with toxic changes as score 4. Lymphocytes were classified as score 0 for normal morphology and score 1 for reactive morphology. The morphological criteria and corresponding scores are described in Table 3. For neutrophils, the animal-level score corresponded to the highest-severity morphological alteration identified among the 100 leukocytes evaluated per animal.

To qualitatively verify interobserver consistency, 100 blood smears (approximately 15% of the total samples) were randomly selected from the four farms in proportion to the number of animals sampled at each farm and independently evaluated by a second assessorexperienced in leukocyte morphology assessment, who was blinded to the first assessor’s results and to the farm and production system. The assessments were compared descriptively; no formal interobserver agreement coefficient was calculated.

#### 2.5.5. Determination of Neutrophil to Lymphocyte (N/L) Ratio

The neutrophil-to-lymphocyte (N/L) ratio was calculated for each animal, by dividing the absolute neutrophil count by the absolute lymphocyte count [42,46].

### 2.6. Statistical Analysis

The individual animal was considered the observational unit, with animals grouped within four farms. Production system (intensive or extensive) was the primary factor of interest. However, farm-specific effects could not be reliably separated from production system and season because only one farm represented each system-by-season combination. Data collection was conducted during two seasonal periods, with both production systems represented in autumn and spring.

Descriptive statistics were used to characterize the main variables observed per farm, including environmental indicators, behavioral indicators, body condition score, cleanliness of water points and animals, health indicators, hematological parameters, differential leukocyte counts, neutrophil-to-lymphocyte ratio, and leukocyte morphology classifications.

Associations between production system and ocular discharge, nasal discharge, coughing, and swelling were explored using the Chi-square test of association, with *p*-values calculated using simulation methods due to low expected frequencies. Because these analyses did not account for farm-level clustering or season, their results were interpreted descriptively and were not considered confirmatory evidence of differences between production systems. For the QBA, a heatmap with hierarchical clustering was generated to visualize behavioral descriptor scores and similarity patterns among farms. Hierarchical clustering grouped farms and behavioral descriptors according to similarities in their QBA profiles. These analyses were descriptive.

Hematological outcomes were additionally explored using generalized estimating equation (GEE) models, including production system (extensive or intensive), season (autumn or spring), and their interaction as explanatory variables. The system-by-season interaction was included to assess whether differences between production systems varied according to season. Gaussian models with an identity link were used for continuous outcomes, whereas binomial models with a logit link were used for binary outcomes. Robust sandwich standard errors were calculated. Adjusted marginal means or probabilities and their respective 95% confidence intervals were estimated from the fitted models, and comparisons between production systems were performed separately within each season using model-based contrasts. However, because each system-by-season combination was represented by only one farm, these model-based results were considered exploratory and were not interpreted as confirmatory evidence of differences attributable to production system or season.

Results are presented as descriptive bar graphs, violin plots with embedded box plots, and exploratory model-adjusted estimates with 95% confidence intervals. All analyses were performed using R software, version 4.3.1 (R Foundation for Statistical Computing, Vienna, Austria) [47].

## 3. Results

### 3.1. Behavioral Assessment

#### 3.1.1. Avoidance Distance Test

**Figure 2:**
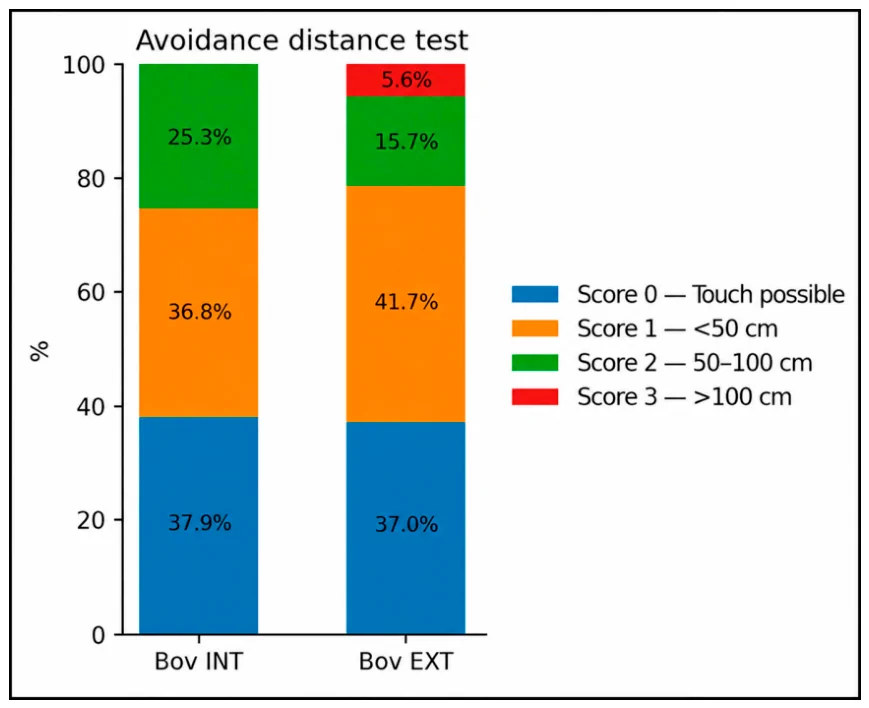
Distribution of avoidance distance test categories in beef cattle from intensive (Bov INT) and extensive (Bov EXT) production systems, considering the animal as the observational unit.

In the intensive system, 37.9% of the evaluated animals were classified as score 0 (touch possible), 36.8% as score 1 (avoidance distance less than 50 cm, without physical contact), and 25.3% as score 2 (avoidance distance between 50 and 100 cm); no animals were classified as score 3. In the extensive system, 37.0% of the animals were classified as score 0, 41.7% as score 1, 15.7% as score 2, and 5.6% as score 3 (avoidance distance greater than 100 cm) (Figure 2).

#### 3.1.2. Cattle Affective States

**Figure 3:**
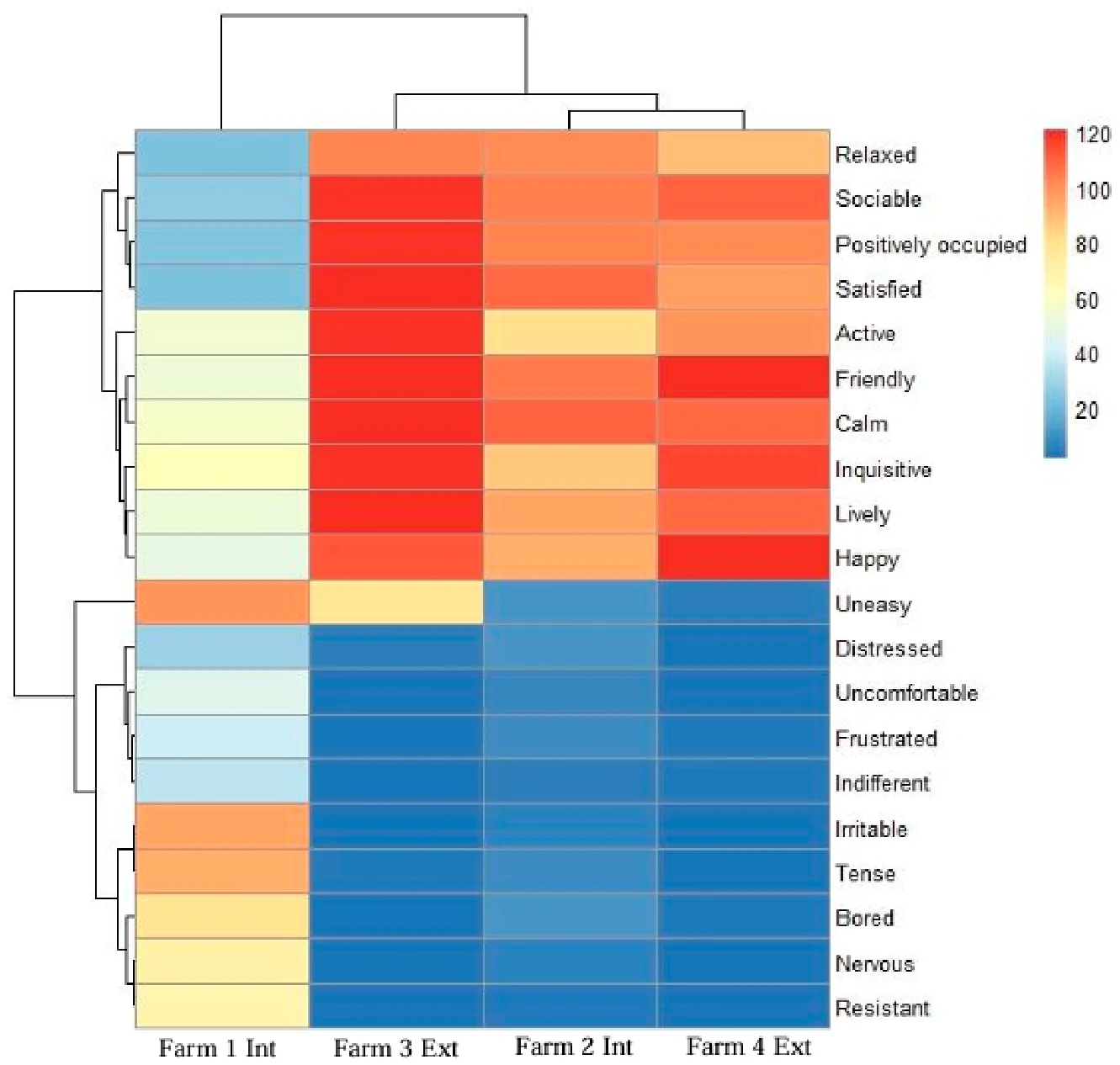
Heatmap of Qualitative Behaviour Assessment (QBA) scores for beef cattle from four intensive and extensive farms located in the north-central, eastern, and central regions of Mato Grosso do Sul, Brazil. Twenty behavioral expressions were assessed using visual analogue scales (VAS), following the Welfare Quality^®^ protocol [40]. The color bar on the right represents the VAS scores, with blue indicating the absence of a behavioral expression and red indicating its maximum perceived expression. The dendrograms illustrate the hierarchical clustering of behavioral expressions (Y-axis) and farms (X-axis), allowing visualization of similarity patterns in QBA profiles. The order of farms on the X-axis was determined by hierarchical clustering based on similarities in their behavioral expression patterns.

The QBA heatmap (Figure 3) showed that three of the four farms exhibited higher scores for positive behavioral expressions and lower scores for negative expressions. In contrast, Farm 1, under an intensive production system, showed a more heterogeneous profile, without a clear predominance of positive behavioral expressions. These descriptive patterns indicate variation in QBA profiles among farms.

### 3.2. Environment, Nutrition and Health Assessment

All animals observed exhibited a normal body condition score (100%), with no cases of severe change detected. Regarding the cleanliness of water points, none of the assessed water points were classified as score 0 (clean). Water points on Farms 2 and 4 were classified as score 1 (partly dirty), with dirty drinkers but fresh and clean water, whereas those on Farms 1 and 3 were classified as score 2 (dirty), with both drinkers and water visibly dirty at the time of inspection.

As for animal cleanliness, most animals on Farms 2 (91.6%), 3 (100%), and 4 (99.3%) were classified as normal, meaning that less than 25% of the body surface was covered with plaques or less than 50% was covered with liquid dirt. On Farm 1, however, only 25% of the animals were classified as normal, while 75% were classified as severe (>25% of the body surface was covered with plaques, or >50% was covered with liquid dirt (Table 4).

**Figure 4:**
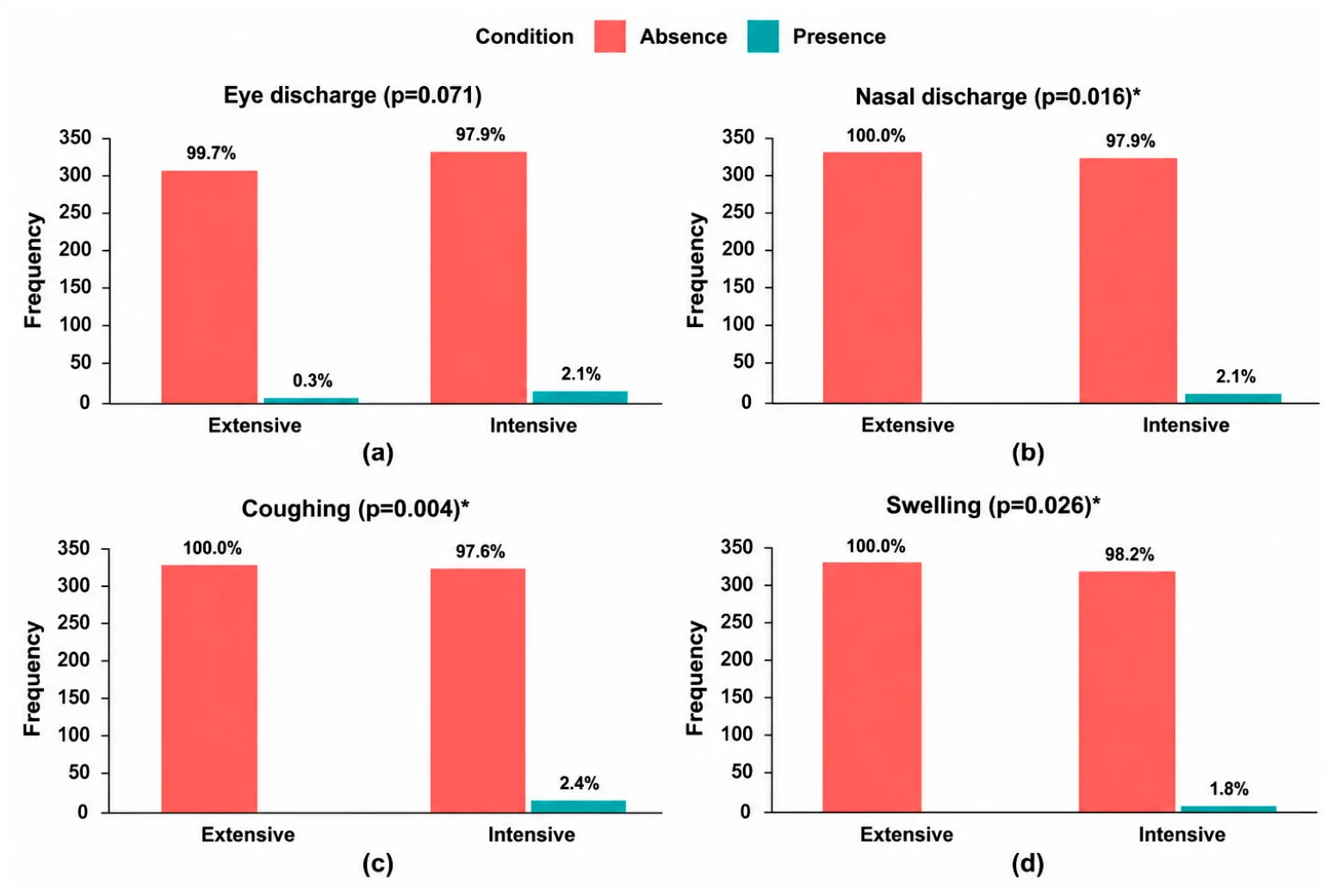
Descriptive frequencies of eye discharge (**a**), nasal discharge (**b**), coughing (**c**), and swelling (**d**) in beef cattle from intensive and extensive systems in farms located in the north-central, eastern, and central regions of Mato Grosso do Sul, Brazil. An asterisk (*) indicates a *p*-value < 0.05 in the unadjusted exploratory Chi-square tests of association, which did not account for farm or season.

The associations between production system and ocular discharge, nasal discharge, coughing and swelling were examined using unadjusted analyses (Figure 4a–d). Nasal discharge was absent in all animals from the extensive system (100%; n = 324), whereas 2.1% (n = 7) of animals from the intensive system presented nasal discharge (score 2; *p* = 0.016) (Figure 4b). Similarly, coughing was absent in all animals from the extensive system, while 2.4% (n = 8) of animals from the intensive system presented coughing (score 2; *p* = 0.004) (Figure 4c). Swelling was absent in all animals from the extensive system, whereas 1.8% of animals from the intensive system presented swelling, particularly in the prepuce region (*p* = 0.026) (Figure 4d). For ocular discharge, 99.7% of animals from the extensive system and 97.9% from the intensive system were classified as score 0 (absence of ocular discharge). Ocular discharge was observed in 2.1% of animals from the intensive system, with no statistically significant association detected in the unadjusted analysis (*p* = 0.071) (Figure 4a).

Regarding the other health indicators assessed, lameness was observed in 11.41% of cattle in the intensive system and was not observed in the extensive system. Hairless spots and lesions were not observed in either system. Difficult breathing and diarrhoea were observed in 5.78% and 2.66% of cattle in the intensive system, respectively, and were not observed in the extensive system. Bloated rumen was not observed in either system.

### 3.3. Differential Counts, Cell Morphology and N/L Ratio

Table 5 presents the descriptive hematological parameters, including hematocrit, total and differential leukocyte counts, and the neutrophil-to-lymphocyte (N/L) ratio, for cattle raised under intensive (n = 340) and extensive (n = 324) production systems. Values are summarized as means and standard deviations, without adjustment for season. Reference intervals reported in [48,49] were used for descriptive interpretation. Season-specific comparisons of neutrophil and lymphocyte counts, N/L ratio, and leukocyte morphology were performed using GEE models.

The descriptive hematological data (Table 5) showed mean hematocrit values of 35.7 ± 6.1% in the intensive system and 34.4 ± 6.1% in the extensive system, both within the reference interval (24–39%). Total leukocyte counts were 8.48 ± 2.39 and 8.07 ± 2.10 × 10^3^/µL, respectively. Band neutrophils were recorded in cattle from the intensive system (0.13 ± 0.28 × 10^3^/µL), whereas none were observed in the extensive system. Metamyelocytes and myelocytes were not detected in either system. Mean eosinophil and monocyte counts were 0.63 ± 0.31 and 0.32 ± 0.32 × 10^3^/µL, respectively, in the intensive system, compared with 0.79 ± 0.40 and 0.47 ± 0.27 × 10^3^/µL in the extensive system.

In autumn, the exploratory GEE estimates indicated higher neutrophil counts in cattle from the intensive system (3.41 [95% CI: 3.24–3.57] vs. 1.81 [1.72–1.90] × 10^3^/µL; *p* < 0.001) and a higher N/L ratio (0.89 [0.84–0.95] vs. 0.38 [0.36–0.39]; *p* < 0.001) compared with cattle from the extensive system. Lymphocyte counts were lower in the intensive group (4.02 [3.91–4.13] vs. 4.96 [4.80–5.12] × 10^3^/µL; *p* < 0.001). The estimated probabilities of severe neutrophil morphology (0.27 [0.22–0.32] vs. 0.10 [0.07–0.14]; *p* < 0.001) and atypical lymphocyte morphology (0.22 [0.18–0.27] vs. 0.10 [0.07–0.13]; *p* < 0.001) were also higher in the intensive group. These findings should be interpreted cautiously because farm-specific characteristics could not be disentangled from production system and season.

**Figure 5:**
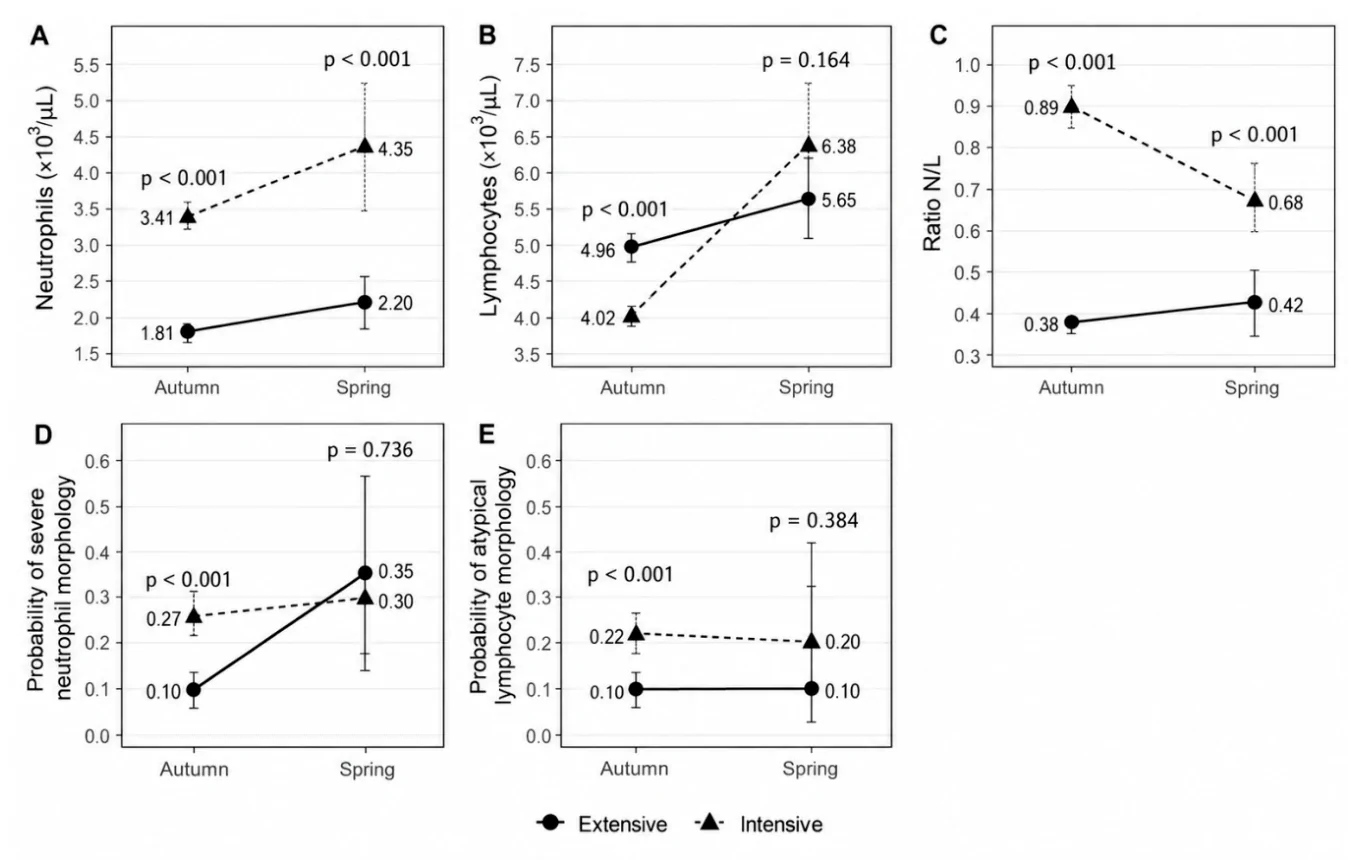
Model-adjusted estimates of hematological parameters and leukocyte morphology in beef cattle from intensive and extensive production systems during autumn and spring in Mato Grosso do Sul, Brazil. Panels show neutrophil counts (**A**), lymphocyte counts (**B**), neutrophil-to-lymphocyte (N/L) ratio (**C**), estimated probability of severe neutrophil morphology (**D**), and estimated probability of atypical lymphocyte morphology (**E**). Estimates were obtained using generalized estimating equation (GEE) models including production system, season, and their interaction. Points represent adjusted marginal means or probabilities, and error bars indicate 95% confidence intervals. *p*-values represent comparisons between production systems within each season.

In spring, the exploratory GEE estimates indicated higher neutrophil counts in cattle from the intensive system (4.35 [95% CI: 3.46–5.24] vs. 2.20 [1.84–2.57] × 10^3^/µL; *p* < 0.001), as well as a higher N/L ratio (0.68 [0.59–0.76] vs. 0.42 [0.33–0.51]; *p* < 0.001). For lymphocyte counts (6.38 [5.51–7.25] vs. 5.65 [5.10–6.20] × 10^3^/µL; *p* = 0.164), severe neutrophil morphology (0.30 [0.14–0.53] vs. 0.35 [0.18–0.58]; *p* = 0.736), and atypical lymphocyte morphology (0.20 [0.08–0.43] vs. 0.10 [0.03–0.32]; *p* = 0.384), the model-based comparisons provided less evidence of separation between the sampled groups. These results should be interpreted prudently because farm-specific characteristics could not be disentangled from production system and season. The season-specific GEE estimates for neutrophil and lymphocyte counts, N/L ratio, and leukocyte morphology are presented in Figure 5.

**Figure 6:**
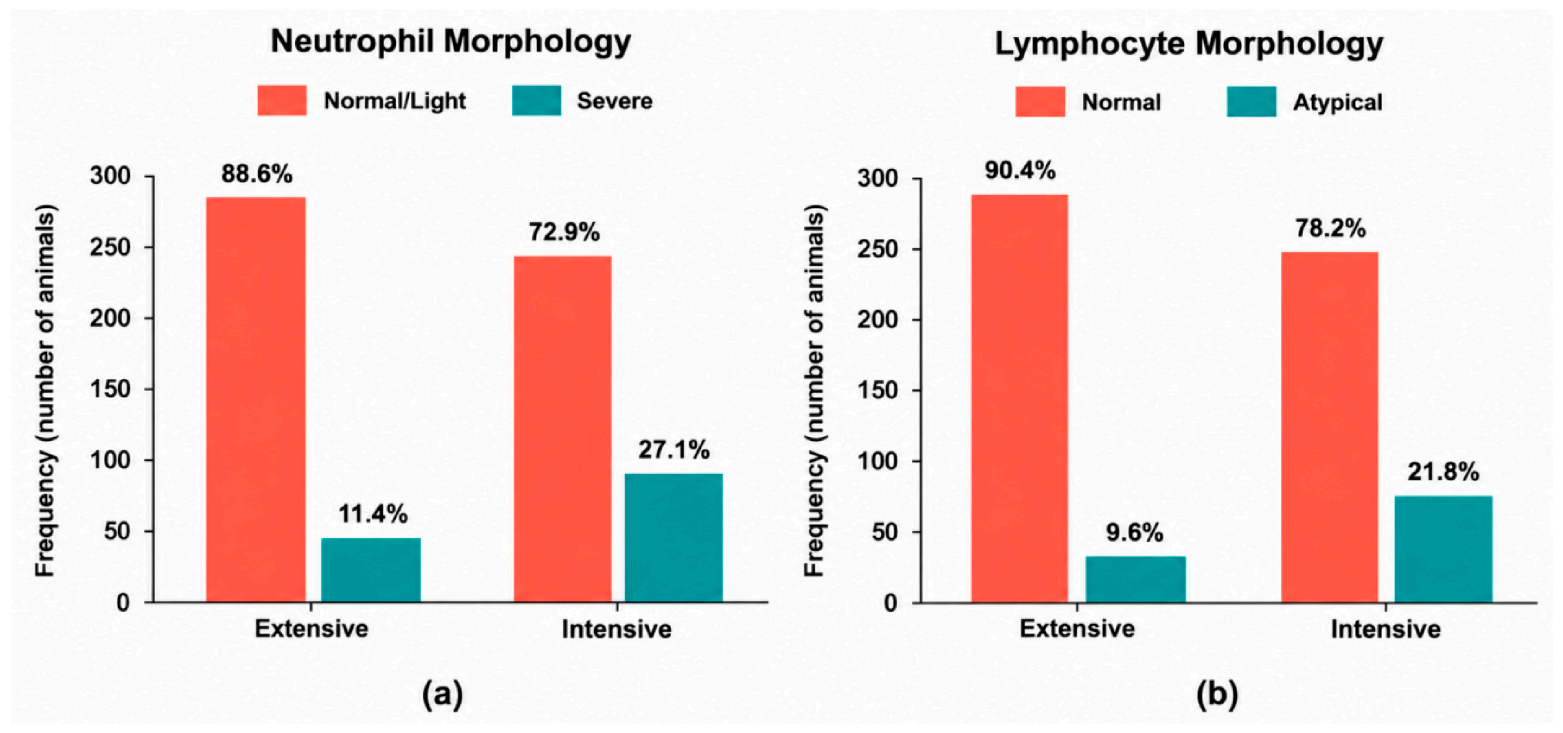
Descriptive frequency distribution of morphological alterations in neutrophils and lymphocytes of beef cattle from intensive and extensive production systems in Mato Grosso do Sul, Brazil. (**a**) Neutrophil morphology, classified as normal/light (scores 0–1) or severe (scores 3–4); and (**b**) lymphocyte morphology, classified as normal (score 0) or atypical (score 1). No animals presented neutrophil morphological changes classified as score 2. Percentages represent pooled observations across autumn and spring, without adjustment for season. Season-specific model-adjusted comparisons are presented in Figure 5.

**Figure 7:**
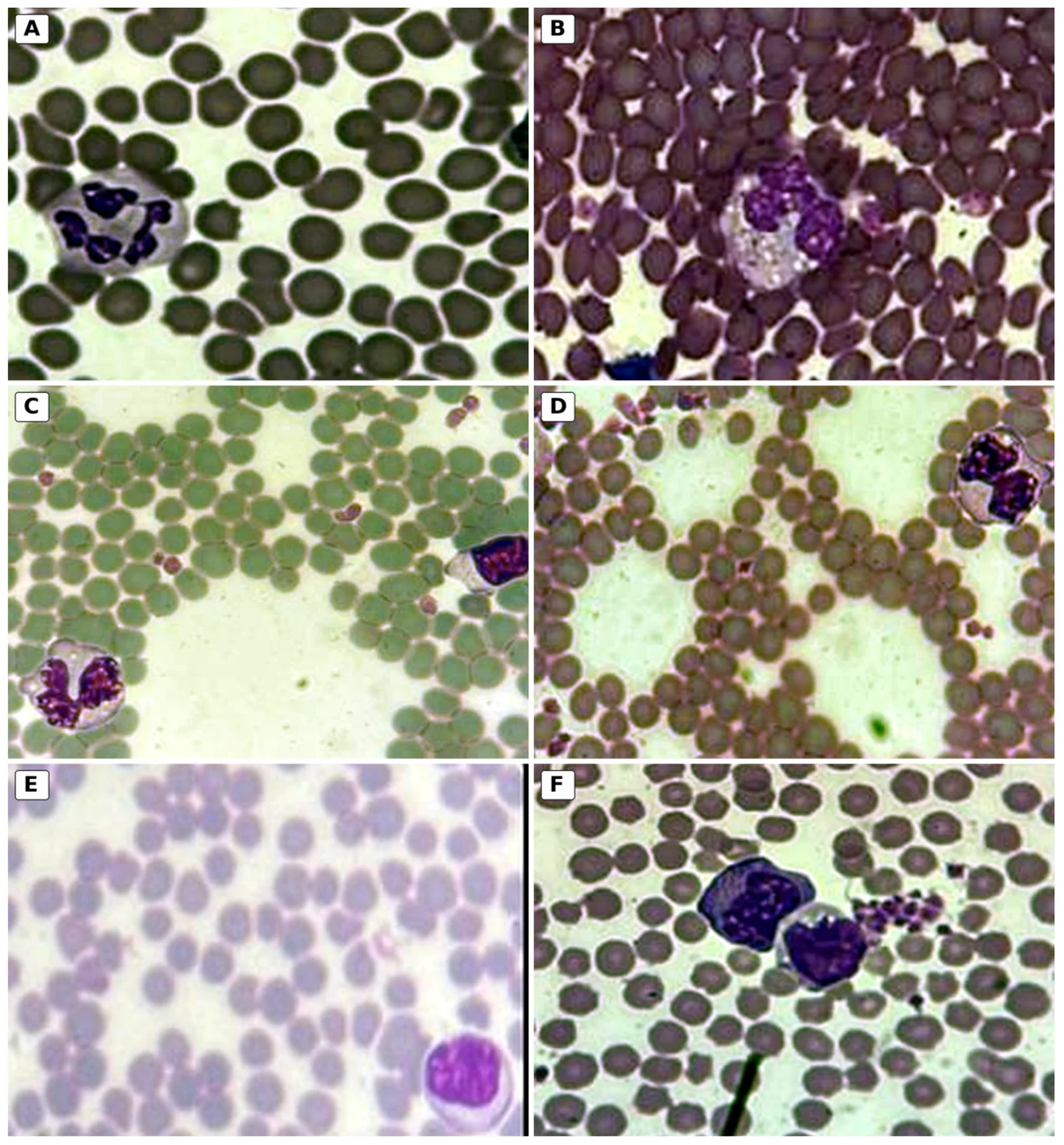
Representative neutrophil and lymphocyte morphology illustrating the leukocyte cell scoring system developed in this study. Peripheral blood smears were obtained from crossbred cattle (Bos taurus indicus × Bos taurus taurus) raised on farms in the North-central, Eastern, and Central regions of Mato Grosso do Sul, Brazil. (**A**) Normal segmented neutrophil (score 0); (**B**–**D**) neutrophils showing severe toxic changes (score 4); (**E**) normal medium lymphocyte (score 0); (**F**) reactive lymphocyte (score 1). Blood smears were stained with methanolic Wright–Giemsa stain and examined using an Axio Scope A1 optical microscope (Carl Zeiss Microscopy GmbH, Germany) equipped with an Axiocam 503 color camera and ZEN Lite (Blue Edition) imaging software at 1000× magnification.

The descriptive distribution of leukocyte morphological classifications is presented in Figure 6. Severe neutrophil morphological alterations (scores 3–4) were observed in 27.1% (n = 92) of cattle from the intensive system and 11.4% (n = 37) of cattle from the extensive system. No animals were classified as score 2. Regarding lymphocyte morphology, atypical forms (score 1) were observed in 21.8% (n = 74) of cattle from the intensive system and 9.6% (n = 31) of cattle from the extensive system. These percentages represent pooled descriptive frequencies across seasons. Season-specific model-adjusted probabilities are presented in Figure 5. Representative neutrophil and lymphocyte morphological features are illustrated in Figure 7.

## 4. Discussion

This study provides preliminary evidence supporting the potential use of leukocyte morphology as a sensitive, objective, and accessible complementary indicator for assessing welfare in beef cattle. The innovative application of a leukocyte morphology scoring system, designed to evaluate alterations in neutrophils and lymphocytes, enabled the detection of immunological changes in beef cattle raised under distinct farm production conditions, which may be relevant to their welfare status. When integrated with behavioral and health indicators collected on-farm, this approach offered a multidimensional framework for evaluating cattle welfare. The leukocyte alterations observed alongside field-based welfare indicators underscore the potential of this method as a practical and scientifically grounded complementary tool. Our results suggest that further research on leukocyte morphology as a complementary measure and potential proxy for on-farm animal welfare assessment is warranted.

Leukocyte morphological assessments revealed biologically relevant differences between animals raised in intensive versus extensive systems, which may reflect physiological responses to environmental, health, and management-related factors. Neutrophils from intensively housed cattle exhibited a higher proportion of severe alterations (27.1%; n = 92), including toxic vacuolization, granulation, and nuclear abnormalities, which are well-documented features associated with inflammatory responses. In contrast, a lower percentage of cattle raised extensively (11.4%; n = 37) showed comparable alterations. Band cells were detected exclusively in the intensive group (0.13 ± 0.28 × 10^3^/µL), indicating a left shift compatible with inflammatory stimulation [33,48,49]. The toxic neutrophil changes detected are recognized as morphological indicators of inflammatory and immune activation [32,33,48]. Their presence in cattle under confinement suggests that animals in such systems may be coping with elevated physiological stress and immunological burden, potentially associated with high stocking density, limited mobility, and reduced environmental enrichment, which may also contribute to negative affective states [33,39].

Lymphocyte morphology also varied between the evaluated systems. Atypical lymphocytes were found in 21.8% of animals on intensive farms compared to 9.6% in extensive systems. The atypical lymphocyte forms, characterized by nuclear irregularities, increased cell size (generally larger than typical lymphocytes), and abundant basophilic cytoplasm, are often associated with chronic antigenic stimulation and stress-induced immunomodulation [33,48,49]. We propose that immunomodulation, as reflected by changes in leukocyte morphology under different production conditions, may provide relevant complementary information for animal welfare assessment.

The hematological findings in our data provided additional information to complement the morphological assessment. Mean hematocrit values were 35.7 ± 6.1% in cattle from intensive farms and 34.4 ± 6.1% in cattle from extensive farms, both within the reference interval (24–39%). Regarding water-point cleanliness, dirty drinkers and water were observed on Farms 1 and 3, both assessed in spring, whereas partly dirty drinkers were observed on Farms 2 and 4, both assessed in autumn. Thus, the observed differences in water-point cleanliness could not be attributed specifically to the production system.

Total leukocyte counts were slightly higher in intensively raised cattle (8.48 ± 2.39 vs. 8.07 ± 2.10 × 10^3^/µL), although mean values in both systems remained within the reference interval. Notably, neutrophil counts were higher in the intensive group (3.46 ± 1.55 vs. 1.84 ± 0.79 × 10^3^/µL), whereas lymphocytes were higher in the extensive group (5.00 ± 1.42 vs. 4.16 ± 1.25 × 10^3^/µL). The season-specific GEE analysis showed that these differences were more pronounced in autumn, whereas in spring the model-based comparison indicated higher neutrophil counts in the intensive group, without clear evidence of a difference in lymphocyte counts. These findings may reflect variations in immune responses associated with the environmental, health, and management conditions of the evaluated farms, as bovine immune function can be influenced by physiological and external factors [42,50].

The neutrophil-to-lymphocyte (N/L) ratio provided relevant information on physiological responses potentially associated with systemic stress. Cattle from intensive farms exhibited higher mean N/L ratios (0.88 ± 0.48) compared with those from extensive systems (0.38 ± 0.15). The season-specific GEE analysis also indicated higher N/L ratios in the intensive group in both autumn and spring, although these differences cannot be attributed exclusively to the production system. These findings are consistent with previous studies identifying the N/L ratio as a physiological indicator associated with stress responses, although its interpretation requires consideration of other physiological, health, and environmental factors [22,30,35,46]. An analogous indicator for birds, the heterophil-to-lymphocyte (H/L) ratio, has long been recognized as an important physiological indicator of stress and is widely used in avian welfare assessment, although recent evidence suggests that its interpretation should consider biological context, including age and physiological state [51,52,53]. Our work contributes to the recognition of the potential relevance of the N/L ratio as a complementary indicator for cattle welfare assessment and highlights the need for further research on its applicability across different management conditions and other mammalian species, as well as across vertebrate taxa in general.

The lower neutrophil-to-lymphocyte (N/L) ratio observed in beef cattle from extensive systems, compared with the intensive group, raises important considerations regarding the interpretation and context of hematological reference values used in welfare assessments. It is possible that reference intervals for the N/L ratio may not fully account for variations in physiological states associated with different environmental and management conditions, including factors potentially affecting animal welfare. Studies have shown that stress, particularly when prolonged or chronic, can induce neutrophilia and lymphopenia, resulting in an increased neutrophil-to-lymphocyte (N/L) ratio [22,23,54,55,56]. Therefore, the lower N/L values recorded in cattle under extensive systems raise the hypothesis that differences in physiological and welfare conditions may influence the interpretation of this indicator, although these values cannot be considered evidence of a more appropriate physiological baseline without further investigation. These findings highlight the need to consider production system, physiological status, health conditions, and welfare context when interpreting hematological reference values and immune-based indicators in cattle [26,55,57,58]. Such an approach may improve the accuracy, biological relevance, and context sensitivity of animal welfare assessments based on hematological parameters.

Descriptive data showed slightly higher monocyte and eosinophil counts in extensively raised cattle compared with those from intensive systems, although these differences alone do not indicate a more stable immune regulatory environment. Chronic stress can disrupt hematopoiesis and affect the functional capacity of these immune cell populations [55,57].

Our experimental design produced results enabling the recognition of the potential relevance of leukocyte morphologyas welfare indicators, since immunological results were investigated in light of the traditional on-field animal welfare assessment. Behavioral and health indicators provided complementary information to the immunological findings. We assessed the human–animal relationship using the avoidance distance test, which may reflect animals’ emotional states and their habituation to human presence [59]. Higher percentages of animals classified as normal, indicating greater tolerance of human proximity, were observed at Farm 2 (39.69%) and Farm 4 (39.66%). In contrast, Farms 3 (14.71%) and 1 (10%) showed lower normal classifications, suggesting differences in responses to human approach. At Farms 1 and 3, the predominance of light behavioral change (40.0% and 52.9%, respectively) indicated mild reluctance to human approach. Farm 1 also showed the highest percentage of moderate change (50.0%), pointing to a more pronounced avoidance. Severe avoidance was recorded only at Farm 3 (14.7%) and Farm 4 (4.5%). The results align with previous studies showing that handling practices, inconsistent interactions, or negative experiences can impair the human-animal relationship, increasing avoidance distances [59,60,61,62]. Conversely, systems promoting gentle and consistent human contact foster shorter distances and more positive human-animal interactions [62,63,64]. Thus, the variation in avoidance distance observed among farms may reflect differences in human-animal interaction quality and broader welfare conditions, rather than a consistent effect of production system, highlighting the importance of behavioral measures alongside physiological and health indicators in comprehensive welfare assessments.

Qualitative Behavior Assessment (Figure 3) revealed a perception that cattle in the extensive system exhibited predominantly positive affective states, indicated by a concentration of observations in the upper red area and a lower frequency of negative states represented in the lower blue region. This pattern suggests a predominance of relaxed, sociable, and environmentally engaged behaviors among the evaluated animals. Conversely, cattle from the intensive system at Farm 1 displayed a distinct behavioral profile, characterized by greater dispersion of emotional responses and the absence of a clear predominance of positive states, suggesting increased variability and a higher occurrence of behaviors associated with discomfort and stress, such as tension, frustration, and resistance. These findings are consistent with existing literature indicating that intensive systems, characterized by movement restrictions, high stocking densities, and limited opportunities for natural behaviors, may be associated with negative emotional states in cattle [34,36]. A surprising aspect of our results indicates the need for further investigation, as Farm 2 presented QBA results closer to Farm 4 than to Farm 1, suggesting variability in behavioral profiles even between farms under the same production system. However, such results need confirmation, as our experimental design does not allow conclusions regarding QBA patterns within or between production systems.

Exploratory statistical analysis indicated associations between production system and the health indicators nasal discharge, coughing, and swelling, but not ocular discharge (Figure 4a–d). Regarding nasal discharge, none of the cattle from extensive farms exhibited signs of discharge, while 2.1% of cattle from intensive systems were classified with severe nasal discharge (*p* = 0.016). Similar patterns were observed for coughing, with 100% absence among cattle in extensive systems and 2.4% of individuals from intensive systems presenting coughing (*p* = 0.004). For swelling, no cases were observed in cattle from extensive systems, while 1.8% of cattle from intensive systems showed swelling, particularly involving the prepuce region (*p* = 0.026). These findings are consistent with existing evidence that intensive production systems may present health challenges associated with higher animal density, environmental contamination, and stress-induced immunosuppression [35,38]. Although the percentages of affected animals in intensive systems were relatively low, their occurrence only in the evaluated intensive farms suggests possible differences in health conditions among farms, which cannot be attributed exclusively to the production system.

Overall, the results emphasize that while good body condition can be maintained across diverse systems, hygiene and health-related welfare indicators, particularly those associated with respiratory and integumentary health, may vary according to farm conditions and management practices. These findings align with the long-recognized need for a multifactorial assessment of cattle welfare, integrating behavioral observations with objective health and hygiene indicators [64,65]. Furthermore, the higher occurrence of nasal discharge, coughing, and swelling observed in cattle from the evaluated intensive farms suggests possible health challenges under these conditions, although these findings cannot be attributed exclusively to the production system.

Our results regarding immune cell numbers and morphology in cattle from intensive and extensive systems suggest differences in immune responses under the evaluated conditions, consistent with current knowledge on the close interaction between environmental stressors and bovine immune function [65,66,67]. Early identification of these immune alterations through hematological parameters and leukocyte morphology may support timely management interventions, potentially helping to reduce disease susceptibility and the need for antimicrobial treatments. Such an approach may also contribute to efforts to mitigate the emergence and dissemination of antimicrobial-resistant bacteria (ARB), one of the most relevant challenges for One Health [68].

Although this study involved 664 cattle from four commercial farms, some limitations should be considered when interpreting the results. The inclusion of intensive and extensive systems evaluated in both autumn and spring allowed us to explore leukocyte morphology under different environmental and management conditions. However, as only one farm represented each combination of production system and season, farm-specific effects could not be reliably separated from those associated with system and season. Differences in health management, age, body weight, and environmental conditions may also have contributed to the observed variation. Routine handling and restraint before blood collection may also have influenced stress-responsive hematological variables, particularly leukocyte counts and the N/L ratio. As habituation to handling was not assessed, potential differences in acute responses to restraint between farms could not be determined, and these findings cannot be attributed exclusively to longer-term production conditions. Farm-specific climatic summaries were not available, limiting the assessment of local environmental influences on the observed leukocyte patterns. In addition, leukocyte morphological alterations may reflect different physiological and immunological responses, including inflammation and infection, reinforcing the importance of interpreting these findings alongside behavioral and health indicators. Despite these limitations, the integration of leukocyte morphology with conventional hematological and on-farm welfare indicators provides a relevant exploratory contribution and supports further investigation of this approach in a larger number of farms and under different management conditions.

Altogether, the combination of behavioral observations, health evaluations, hematological parameters, and leukocyte morphology provides a multidimensional approach to assessing cattle welfare, consistent with current recommendations advocating the integration of multiple stress biomarkers to improve the accuracy and biological relevance of welfare assessments [69]. The leukocyte morphology scoring system developed and applied in this study enabled the detection of cellular alterations potentially associated with stress-related immune responses, representing a novel and practical complementary approach to welfare assessment. By integrating cellular morphology into routine hematological evaluation, this method may expand the value of blood smears beyond conventional leukocyte counts and offers new opportunities for investigating leukocyte morphology as an objective complementary indicator of animal welfare.

## 5. Conclusions

Leukocyte morphology showed differences between cattle raised under the intensive and extensive farming conditions evaluated in this study, suggesting its potential as a complementary indicator of animal welfare. Stress responses can induce immunomodulatory and adaptive effects that alter the counts and the morphology of leukocytes. The comprehension of such phenomena in cattle is limited and generally centered on counting leukocytes and the neutrophil-to-lymphocyte ratio to measure stress. Our results further suggest that morphological evaluation complements conventional hematological assessment by providing biologically meaningful information potentially associated with animal welfare. When interpreted together with behavioral observations and health indicators, leukocyte morphology offers a practical, objective, and low-cost approach for multidimensional welfare assessment in beef cattle. In addition, this study shows that routine blood smears can contribute to the understanding of animal welfare. Leukocyte morphology may reflect physiological responses of animals to the environmental, health, and management conditions to which they are exposed, and may expand the biological interpretation of routine hematological assessments, although these alterations are not specific to welfare status. Incorporating this information into welfare assessment may provide an additional tool to support management decisions and promote more ethical, resilient, and sustainable food production systems.

## Figures and Tables

**Table 1:** Main characteristics of the assessed beef cattle farms located in the North-central, Eastern, and Central regions of Mato Grosso do Sul, Brazil.

Variable	Farm 1	Farm 2	Farm 3	Farm 4
Production system	Intensive (Confinement)	Intensive (Confinement)	Extensive (Open pastures)	Extensive (Open pastures)
Municipality	Jaraguari	Campo Grande	Ribas do Rio Pardo	Camapuã
Season	Spring	Autumn	Spring	Autumn
Farm size (ha)	274	387	577	1750
Herd size	700	2500	540	1750
Breed	Nellore Cross	Nellore Cross	Nellore Cross	Nellore Cross
Identification	Ear tags	Ear tags	Ear tags	Ear tags
Age (months)	20 ± 4s	24 ± 4	20 ± 4	24 ± 4
Feedlot space allowance (m^2^/animal)	20	10–15	N/A	N/A
Pasture stocking rate (animals/ha)	N/A	N/A	1	1
Average weight (kg) *	450 ± 40	480 ± 50	400 ± 50	420 ± 40
Feeding strategy	Concentrate 4X/day	Concentrate 4X/day	Pasture and Concentrate 1X/day	Only Pasture
Bunk feeder and size/head (cm)	Plastic barrel 45 × 50	J type Concrete 45 × 50	Plastic barrel 45 × 50	Plastic barrel 45 × 50
Water Trough and size	Plastic barrel 200 L	Tilting water 600–1200 L	Large metal 2600 L	Concrete waterer 690 L

* Individual body weight was not systematically recorded; therefore, the weights presented represent average values for the evaluated groups at each farm.

**Table 2:** Herd health management practices evaluated across intensive and extensive beef cattle production systems in Mato Grosso do Sul, Brazil.

Variable	Farm 1	Farm 2	Farm 3	Farm 4
Vaccination	Clostridiosis	Clostridiosis BVD, IBR, BRSV, PI3, Pasteurellosis, Leptospirosis	Clostridiosis	Clostridiosis
Deworming	Doramectin 3.5%	Albendazole sulfoxide 15%	Albendazole sulfoxide 15%	Ivermectin^®^ 3.5%
Dehorning and castration procedure	N/A	N/A	N/A	N/A
Mortality (%/year)	<1	0	<1	1.5

N/A: not applicable. Pain management was not applicable because dehorning and castration were not performed.

**Table 3:** Description of the morphologic diversity observed on the blood films of cattle neutrophil and lymphocyte cells for their classification, as per morphological criteria described by [32,33,45].

Cell	Description of the Morphologic	Score	Classification	Images
Neutrophil segmented normal	Normal morphology; cytoplasm filled with fine, small granules and with slight acidophilic (pink). Tri or S-shaped segmented nucleus (3 to 5 lobes), connected by thin filaments of dense chromatin (dark blue).	0	Normal	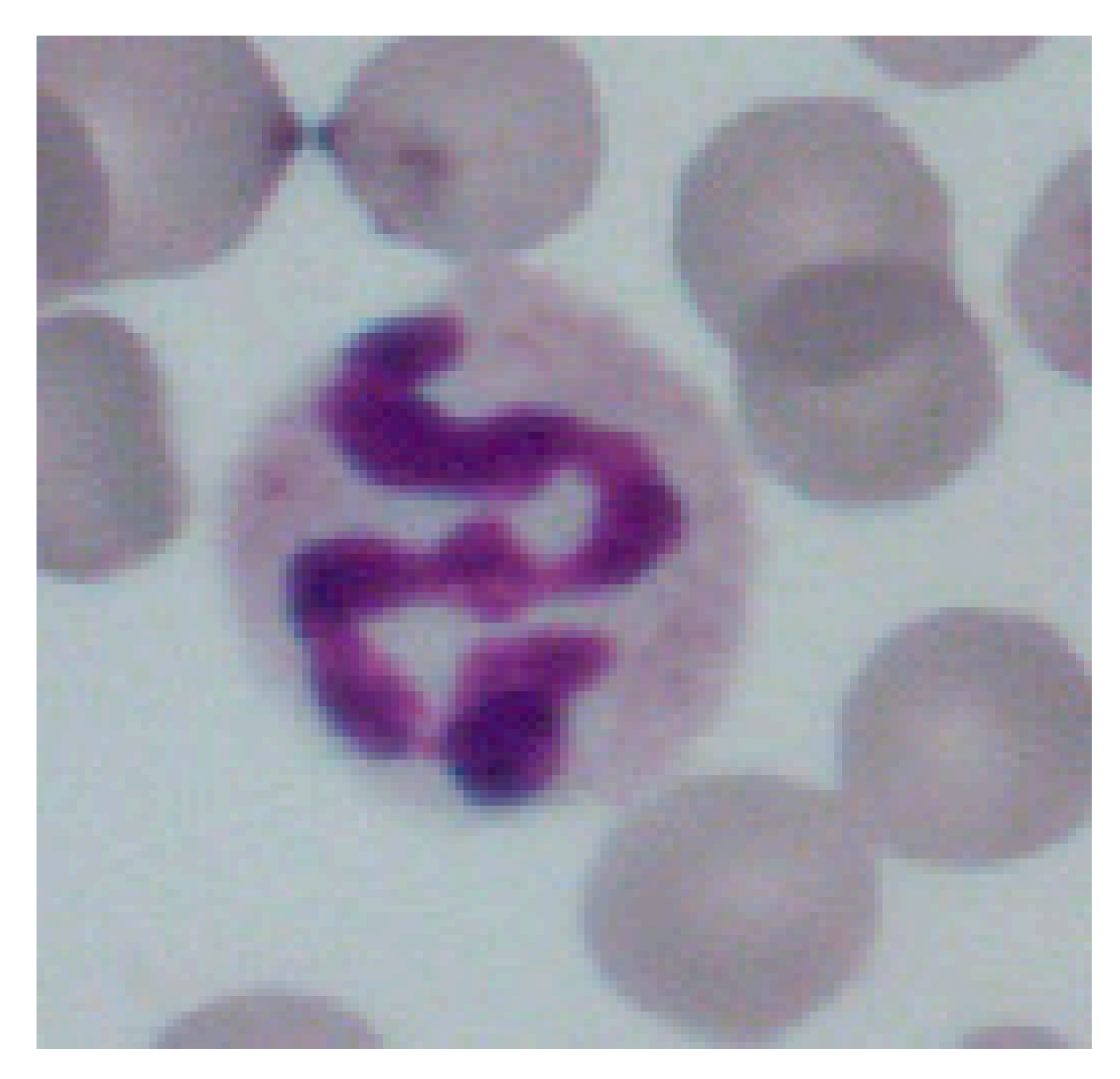
Band Neutrophil	Light change; cytoplasm containing light pink granules; with typical horseshoe shaped nucleus and non-segmented.	1	Light change	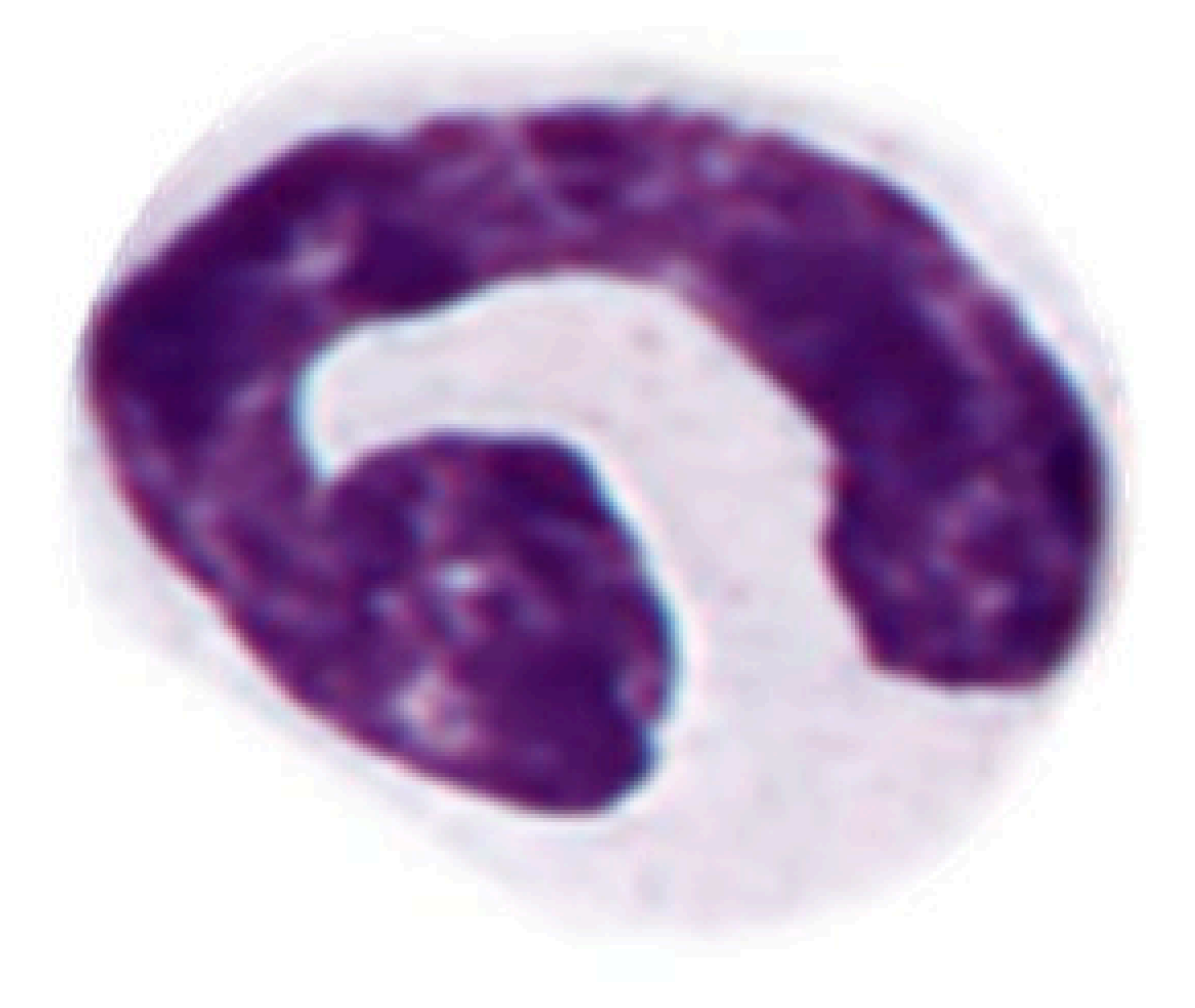
Metamyelocyte (±left shift)	Moderate change; presence of metamyelocyte faint blue cytoplasm and with typical kidney-bean shaped nucleus and less clumped chromatin	2	Moderate change	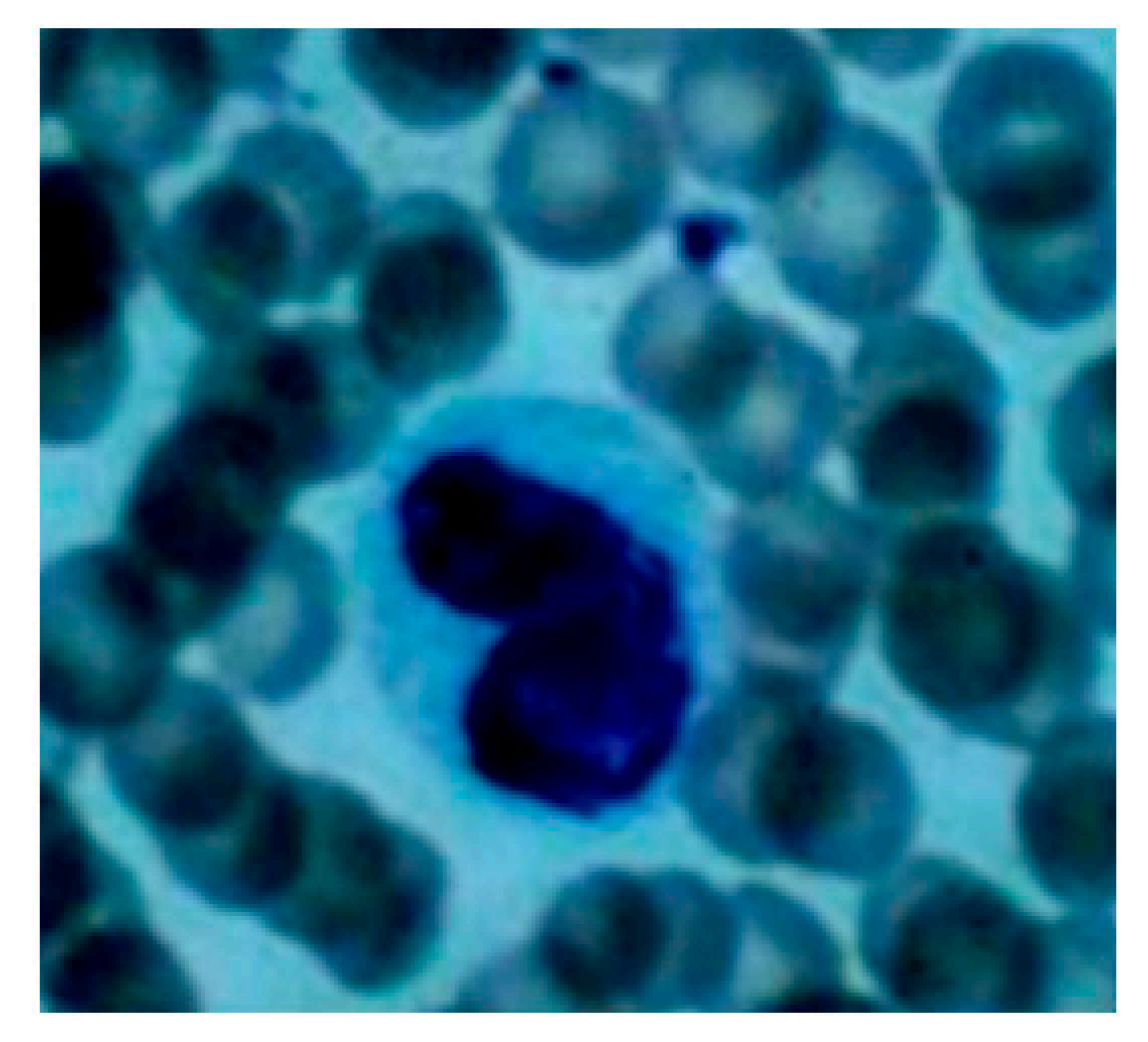
Myelocyte (±left shift)	Severe change; presence of myelocyte faint blue cytoplasm with typical large spherical and nonsegmented nucleus and less clumped chromatin.	3	Severe change	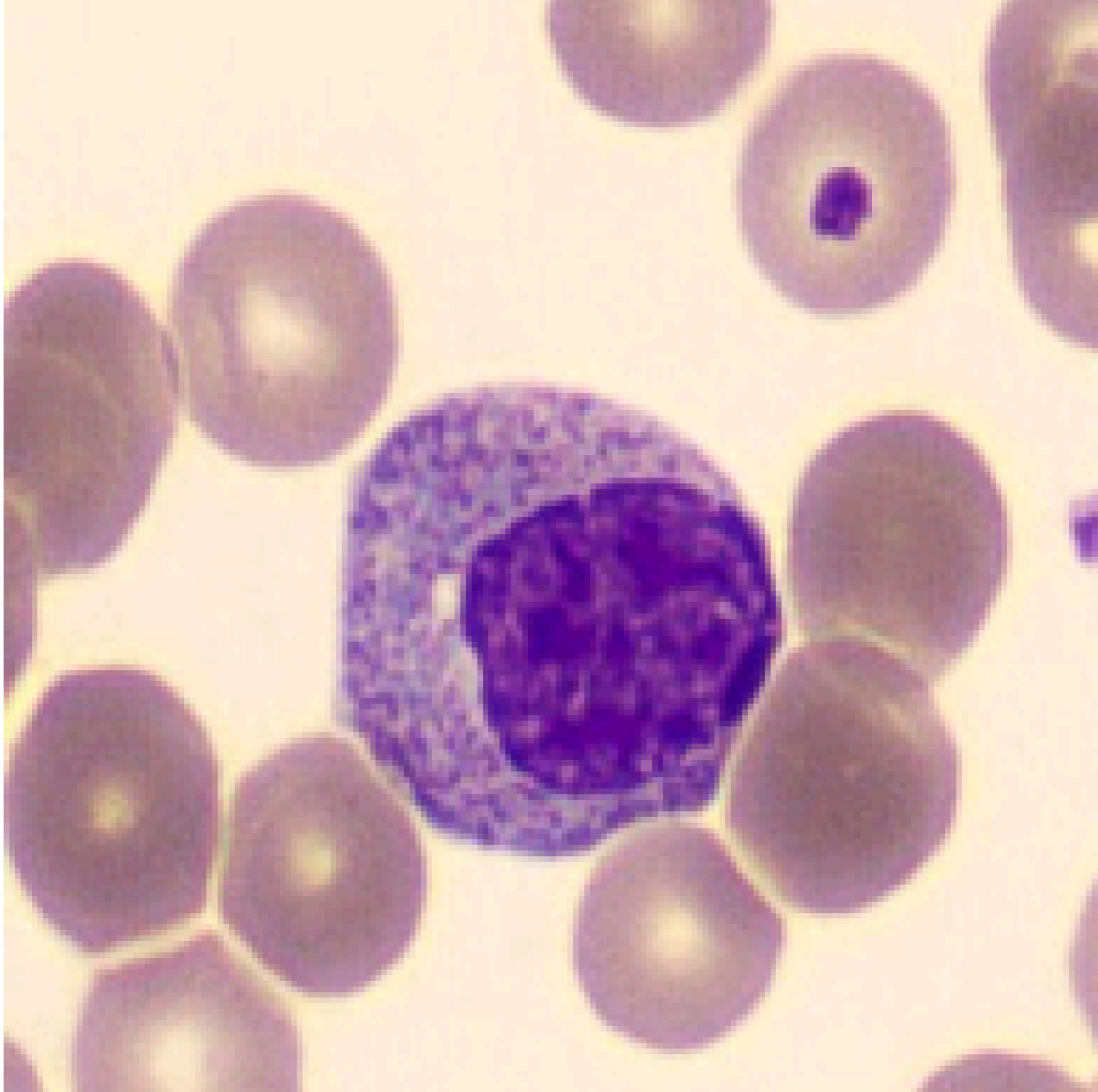
Neutrophil with toxic changes (±left shift)	Severe toxic change; endotoxin induced abnormal PMN (evidence of delayed maturation). Basophilic and azurophilic granules and vacuoles in the cytoplasm. Pale blue inclusions of ribosomes and RNA and left shift (band, metamyelocyte and myelocyte) may be present.	4	Severe change	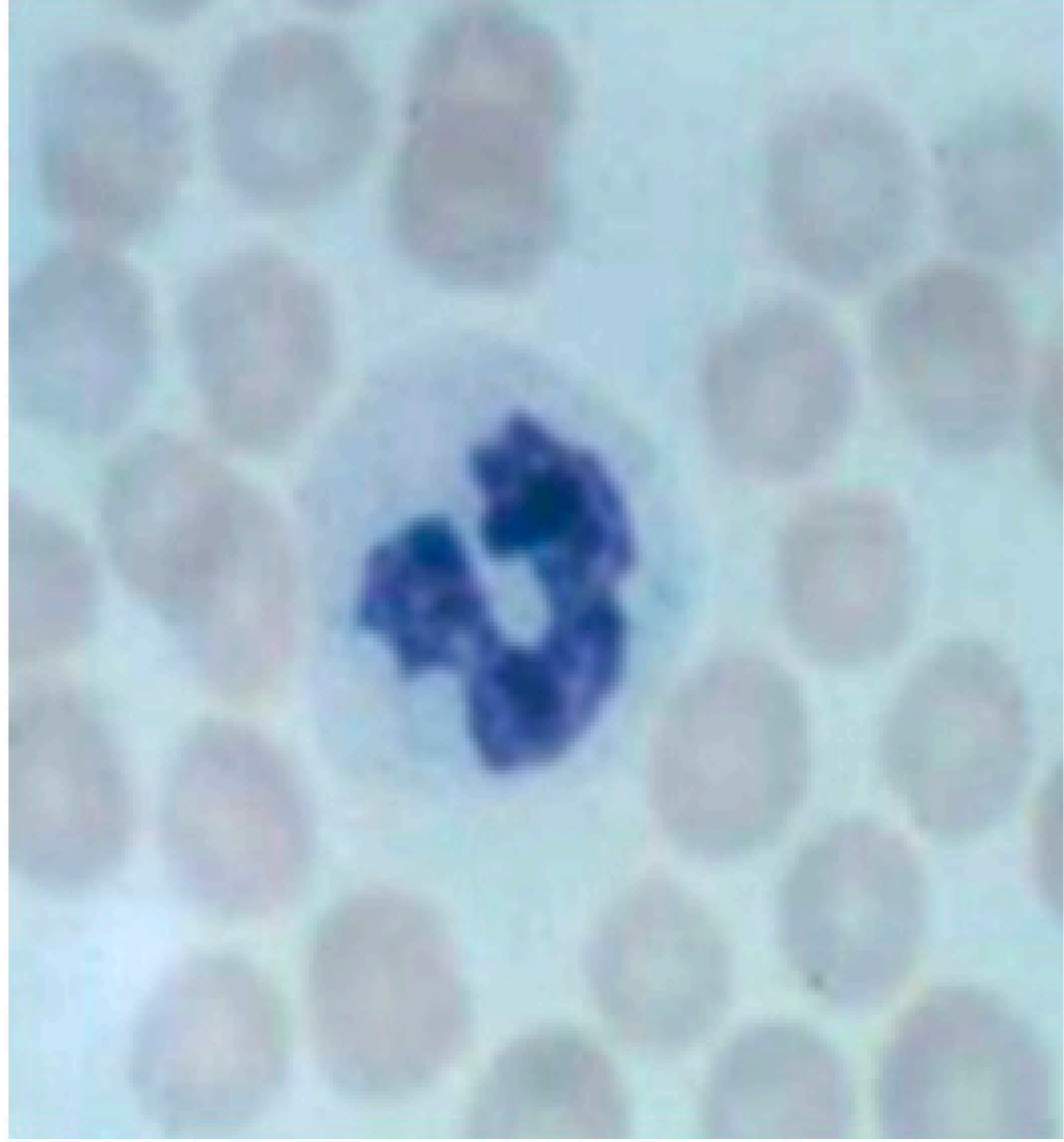
Normal lymphocyte	Normal morphology; small (A) and medium (B) lymphocytes, the cytoplasm forms an extremely narrow, weakly basophilic rim around the nucleus and in some cells, azure granules may be present; circular nucleus with darkly staining with chromatin arranged in dense blocks (dark blue), occupying 70 to 90% of the cytoplasm; large (C) lymphocytes, the cytoplasm is pale blue, faintly basophilic, and frequently contains small vacuoles; nucleus may be round, oval, kidney-shaped, or have a few deep indentations.	0	Normal	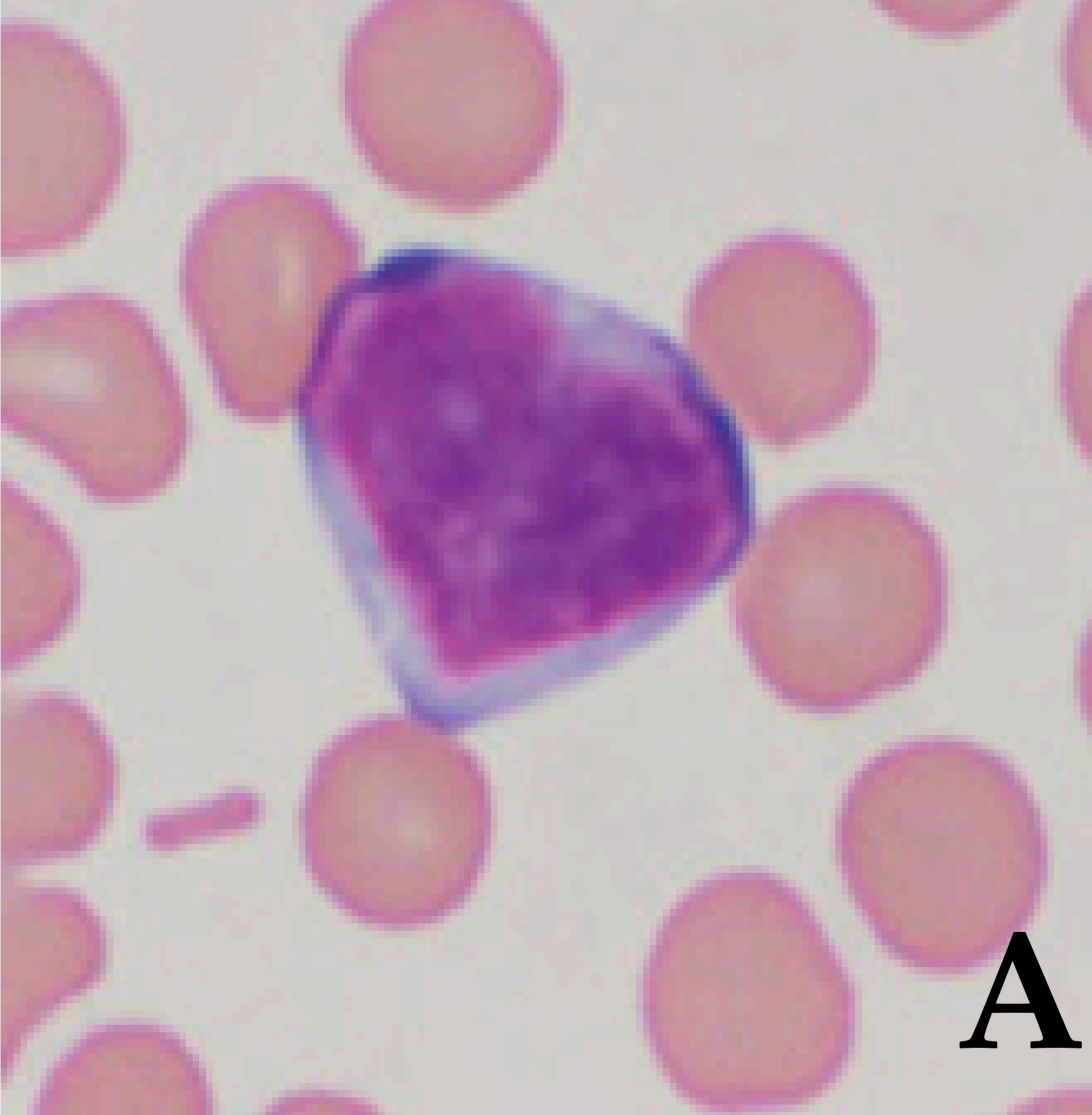 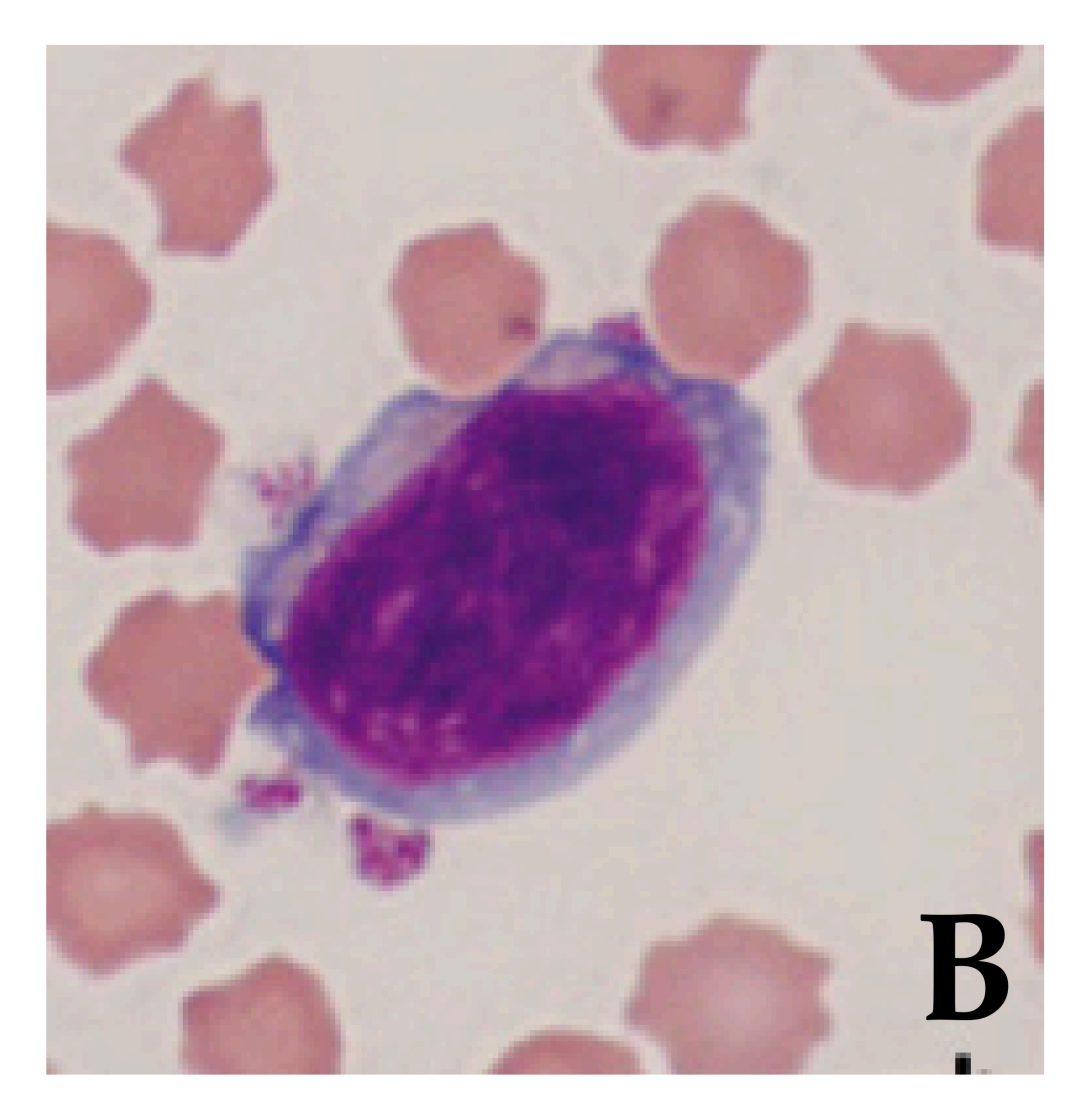 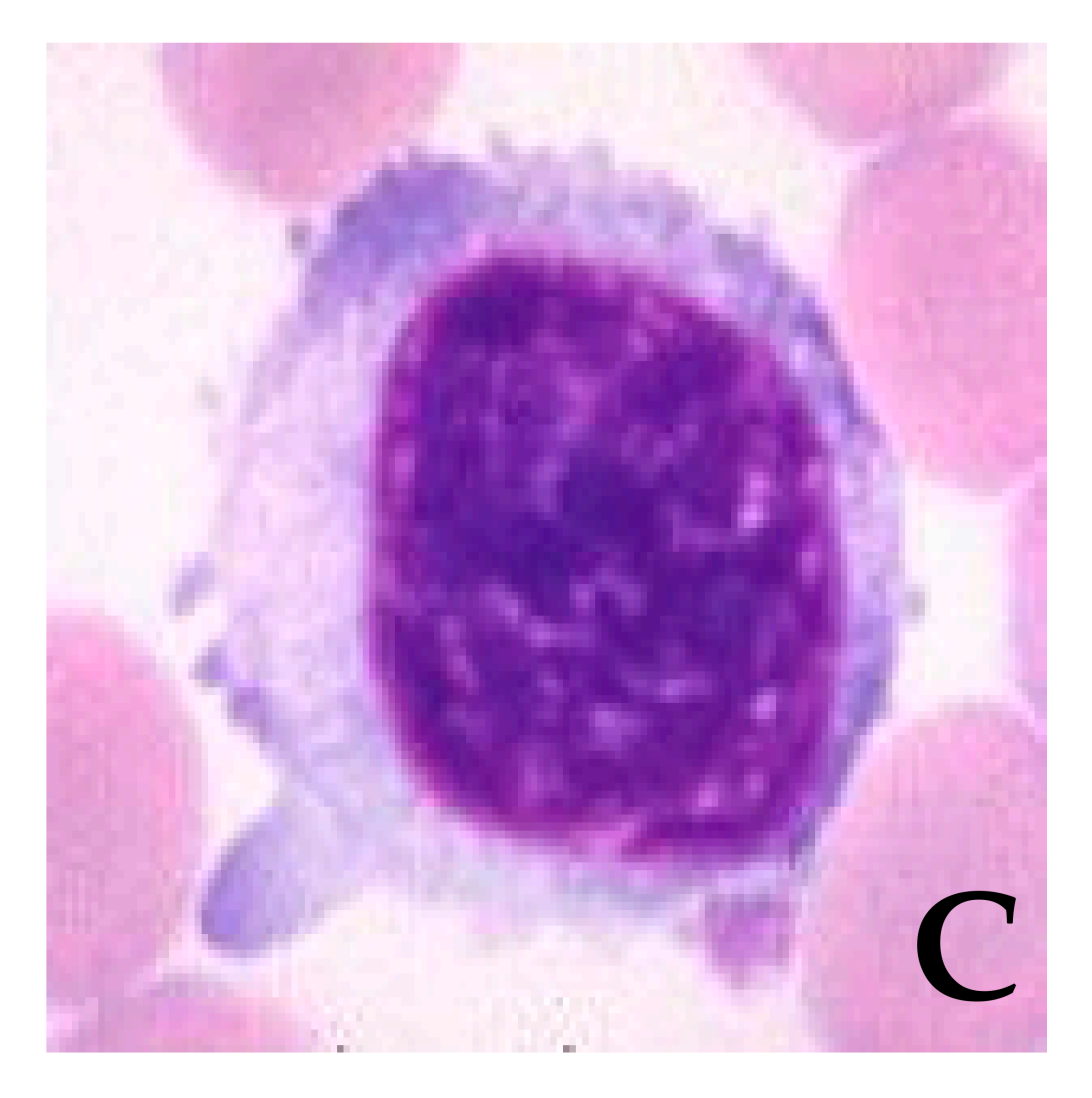
Reactive lymphocyte	Abnormal morphology; reactive lymphocyte, increased cytoplasm with intense basophilia; irregular, scalloped, or cleaved nuclei with clumped chromatin.	1	Abnormal	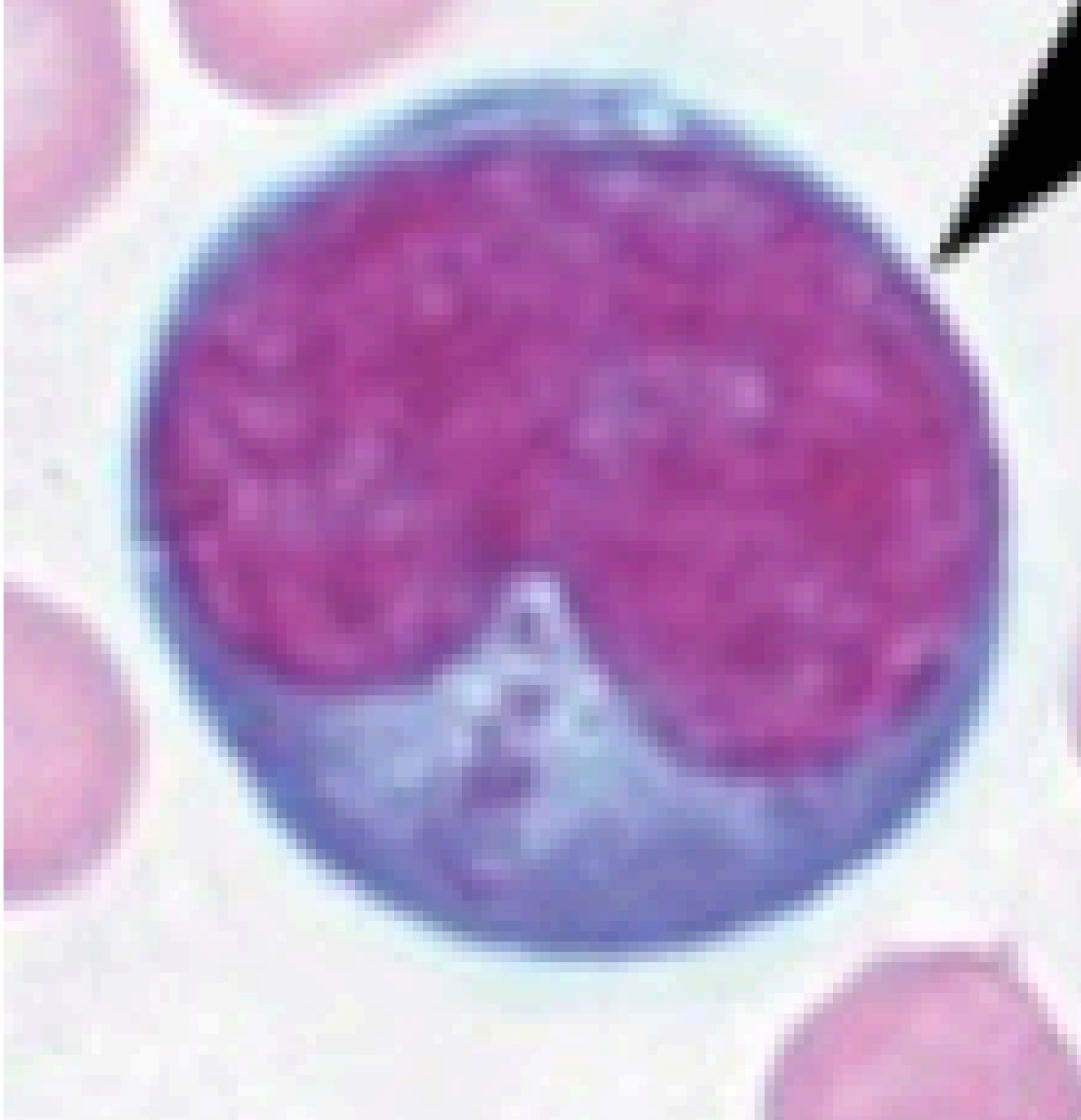

**Table 4:** Classification of nutrition and environmental welfare indicators scores in beef cattle from intensive and extensive systems in farms located in the north-central, eastern, and central regions of Mato Grosso do Sul, Brazil.

Principle	Measures	Classification	Farm 1 Intensive	Farm 2 Intensive	Farm 3 Extensive	Farm 4 Extensive
Good feeding	Body condition score %	Normal	100	100	100	100
		Severe change	0	0	0	0
	Cleanliness of water points %	Clean (score 0)	0	0	0	0
		Partly dirty (score 1)	0	100	0	100
		Dirty (score 2)	100	0	100	0
Good housing	Cleanliness of the animals %	Normal	25	91.6	100	99.3
		Severe change	75	8.4	0	0.7

**Table 5:** Descriptivehematologicalparameters and differential leukocyte counts of 664 beef cattle from intensive (n = 340) and extensive (n = 324) production systems in Mato Grosso do Sul, Brazil. Values are presented as mean ± standard deviation, without adjustment for season.

Variable	Intensive System	Extensive System	Reference Values ^a^
	*N* = 340	*N* = 324	
Hematocrit (%)	35.7 ± 6.1	34.4 ± 6.1	24–39
TLC (×10^3^/μL)	8.48 ± 2.39	8.07 ± 2.10	4.4–10.8
Neutrophil (×10^3^/μL)	3.46 ± 1.55	1.84 ± 0.79	0.8–5.0
Band (×10^3^/μL)	0.13 ± 0.28	0.00	0–0.01
Meta + Mielo (%)	0.00	0.00	0
Lymphocyte (×10^3^/μL)	4.16 ± 1.25	5.00 ± 1.42	1.8–4.9
Eosinophil (×10^3^/μL)	0.63 ± 0.31	0.79 ± 0.4	0.1–2.1
Basophil (×10^3^/μL)	0.00	0.00	0.00
Monocyte (×10^3^/μL)	0.32 ± 0.32	0.47 ± 0.27	0.3–1.2
N/L ratio	0.88 ± 0.48	0.38 ± 0.15	-

^a^ Reference intervals according to [48]; where applicable. TLC = total leukocyte count; Meta = metamyelocytes; Mielo = myelocytes; N/L ratio = neutrophil-to-lymphocyte ratio. Values are descriptive and were not adjusted for season.

## Data Availability

The data presented in this study are available from the corresponding author upon reasonable request. The data are not publicly available because they contain information related to privately owned farms.

## Supplementary Materials

The following supporting information can be downloaded at https://www.mdpi.com/article/10.3390/ani16213356/s1.
