# Supplementary material for: Can Leukocyte Morphology Contribute to Welfare Assessment in Beef Cattle Raised Under Intensive and Extensive Farming Systems?
Source: Animals (Basel). 2026 Oct 24;16(21):3356. doi: 10.3390/ani16213356 (PMC13761867; doi:10.3390/ani16213356)
Supplement: Supplementary file 1 [file animals-16-03356-s001.zip › animals-4527539-supplementary.pdf]

Can Leukopcyte Morphology Contribute to Welfare Assessment in Beef Cattle Raised Under Intensive and Extensive Farming Systems?

Laura Raquel Rios Ribeiro[1*], Juliana do Canto Olegário[1]; Alexandre de Oliveira Bezerra[2], Ricardo Martins Santos[3]; Cesar Augusto Taconeli[4]; Carla Forte Maiolino Molento[1]
Correspondence: laurarrribeiro@hotmail.com

Supplementary Figures S1-S4.

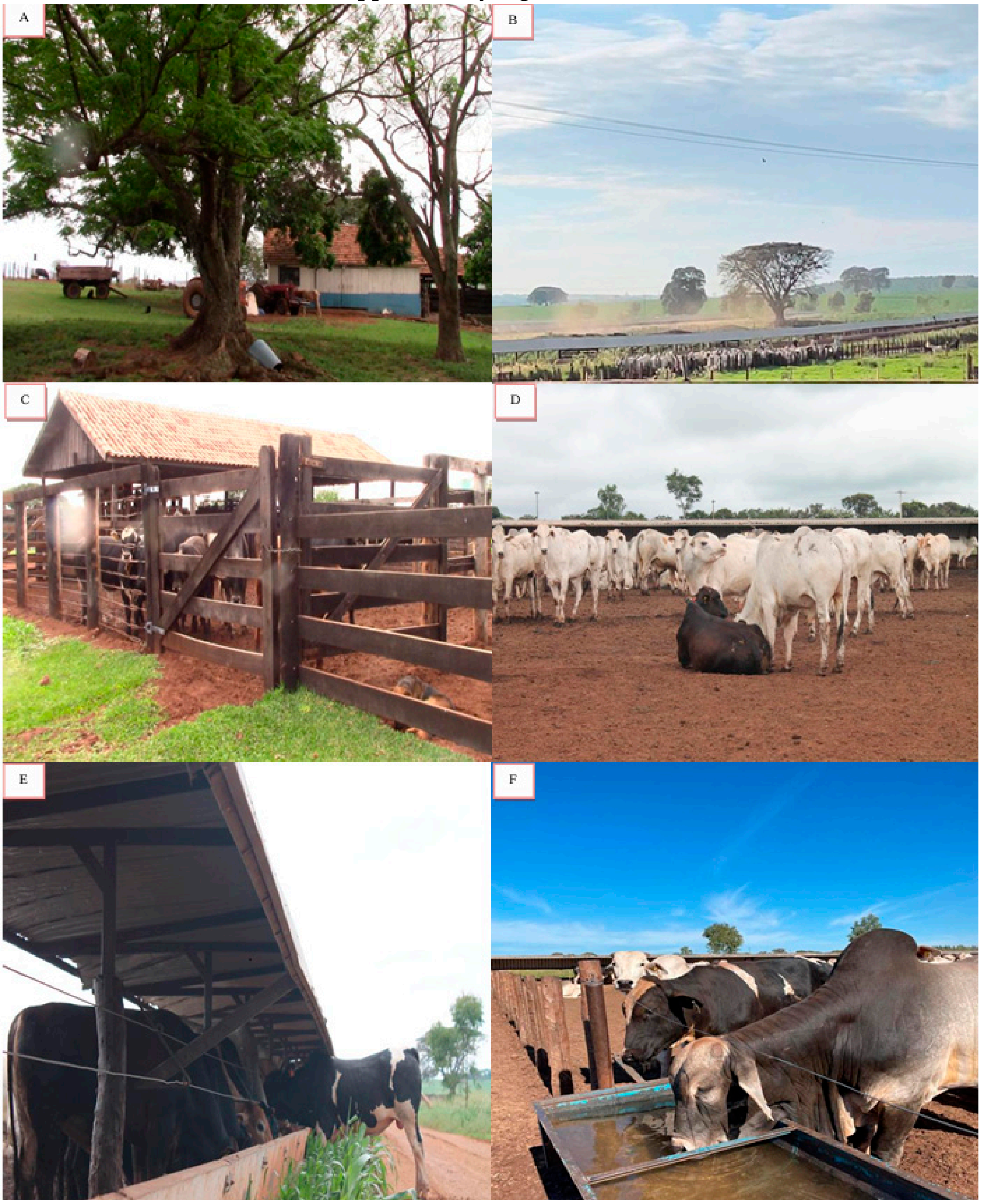

Figure S1. External view of intensive cattle farming on farm 1 (A) and farm 2 (B); on farm 1, cattle were located near the squeeze chute (C); on farm 2, they were housed in adjacent feedlot pens (D); feed bunks in the pens were made of concrete (E) and tilting drinking troughs (F).

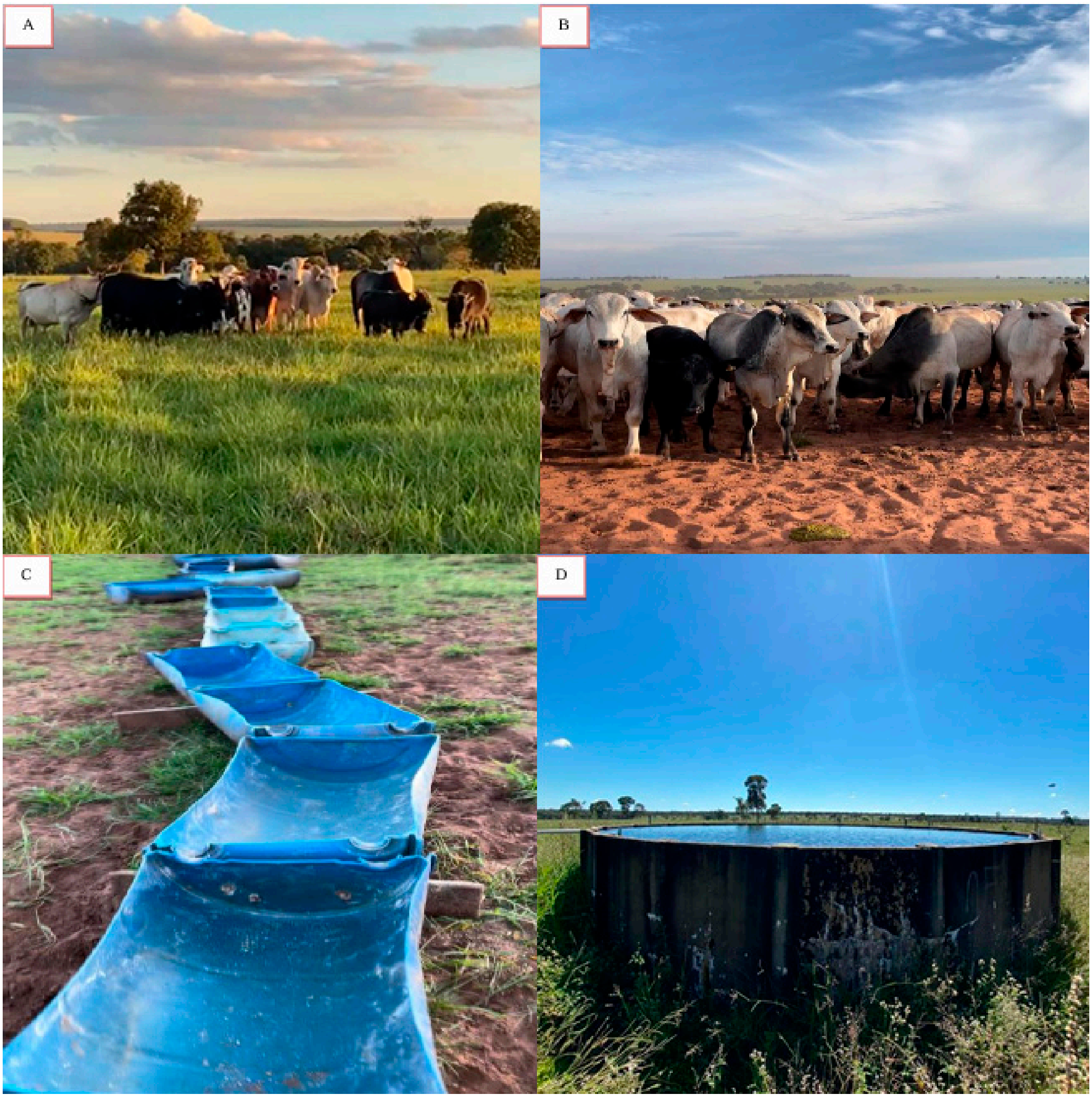

Figure S2. External view of extensive cattle farming at Farm 3 (A) and Farm 4 (B). Farm 3 cattle were raised on pasture with dietary supplementation and received feed in a plastic barrel-type feeder (C), with access to water from an Australian-type large metal trough (D). Farm 4 cattle were raised exclusively on pasture, with water provided in a 690-L concrete waterer.

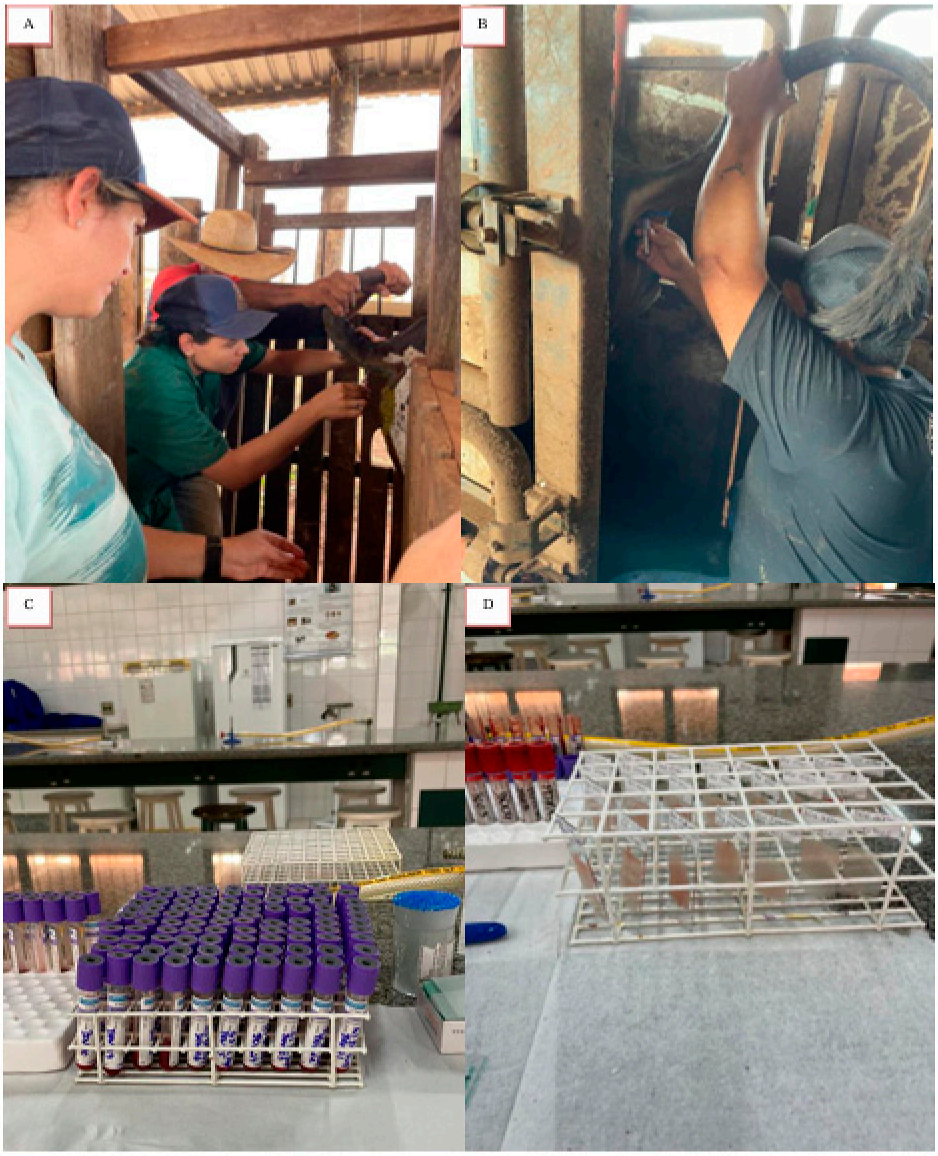

Figure S3 Blood samples collected from the coccygeal vein (A–B) in 664 beef cattle from farms located in the north-central, eastern, and central regions of Mato Grosso do Sul, Brazil; samples were stored in EDTA tubes (C) and then stored in a thermal box and transported to the laboratory for blood smear preparation (D) and subsequent hematological analyses.

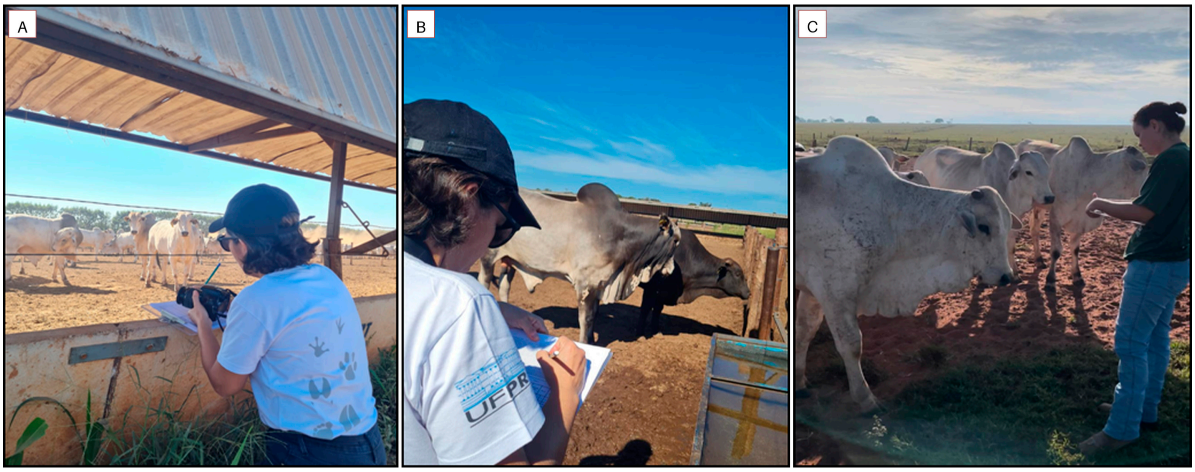

Figure S4. Welfare assessment procedures and production environments observed during field visits. (A) Qualitative Behaviour Assessment (QBA) conducted through direct observation of cattle behaviour. (B) Evaluation of animal- and resource-based indicators according to selected measures adapted from the Welfare Quality® protocol. (C) Avoidance distance test used to assess the human–animal relationship. Photographs were obtained during visits to commercial beef cattle farms in Mato Grosso do Sul, Brazil.

Supplementary Table

Table S1: Principles, criteria and selected measures, methodology and scoring, based on the Welfare Quality® protocol for cattle (Welfare Quality®, 2009).

Principle	Criteria	Measure	Method	Score
Good feeding	Absence of prolonged hunger	Body condition score	View the animal from behind and from the side in the loin, tail head and vertebrae	0 - Normal Satisfactory body condition<br>2 - Very lean
	Absence of prolonged thirst	Cleanliness of water points	All water points are assessed within the area of the animal unit	0 – Clean: drinkers and water clean<br>1 – Partly dirty: drinkers dirty but water fresh and clean<br>2 – Dirty: drinkers and water dirty
Good housing	Comfort around resting	Cleanliness of the animals	Visual inspection of one side of the animal for assessment of the degree of dirt on the body parts	0 – < 25% area with plaques, or < 50% with liquid dirt<br>2 – ≥25% area with plaques, or > 50% with liquid dirt
Good health	Absence of injuries	Lameness	Visual inspection of walking ability	0 – % without lameness<br>2– % with lameness
		Integument alterations	Visual inspection of hind leg, body and front leg.	0 - % without integument alteration<br>2 - % with integument alterations (hairless spots, lesions and swellings)
	Absence of disease	Coughing, Discharge, breathing, diarrhea, rúmen	Count coughs/animal/15’<br>Visual check for signs of nasal and ocular discharge, difficult breathing, diarrhoea and bloated rúmen	0 - % without evidence<br>2 - % with evidence
Appropriate behavior	Good human-animal relationship	Avoidance distance	Approximation of the animal directing the back of the hand toward the	0 – % that allow touch<br>1 – % approachable within <50 cm, but not touchable<br>2 – % approachable within 50–100

		animal and walking until signs of withdrawal occur, or until you can touch the nose/muzzle.	cm<br>3 – % not approachable within 100 cm
	Qualitative behaviour assessment	Eight observation points with varying durations; 10-min acclimation; assess qualitative expression of group behavior and interaction with peers and environment.	20 descriptors using Visual analogue scale
